# Socioeconomic, behavioral, and psychological correlates of body weight and weight gain: evidence from a 12-year German panel study

**DOI:** 10.1186/s12889-026-30138-w

**Published:** 2026-10-31

**Authors:** Felix Bittmann

**Affiliations:** Leibniz Institute for Educational Trajectories (LIfBi), Wilhelmsplatz 4, Bamberg, 96047 Germany

**Keywords:** Obesity, Overweight, Body mass index, Weight gain, Longitudinal study, Personality traits, Physical activity, Germany

## Abstract

**Background:**

Overweight and obesity are major public health challenges in Germany. Body weight is shaped by a wide range of socioeconomic, behavioral, and psychological factors, but less is known about how these factors jointly relate to weight gain among adults in Germany.

**Methods:**

Data were drawn from the adult cohort of the National Educational Panel Study (NEPS-SC6). The analytical sample included 8,209 individuals aged 28–55 years at baseline (2011/12), followed over approximately 12 years. In this exploratory study, cross-sectional associations with BMI and overweight/obesity status were estimated using regression models, while longitudinal associations with BMI changes were analyzed using fixed-effects panel models, capturing within-individual changes. Missing values were handled by multiple imputation (50 imputed datasets).

**Results:**

Higher household income (-0.16), tertiary education (-0.41), higher social origin (-0.26), greater conscientiousness (-0.64), higher neuroticism (-0.27), and more frequent sports participation (-0.38) were associated with lower BMI at baseline, whereas higher extraversion was associated with higher BMI (+ 0.23). Women had significantly lower BMI than men (-1.42). Longitudinal analyses showed that BMI increased over time (0.08 points per year), with the steepest increase in the first three years. Within individuals, increases in household income (+ 0.10) and transitions into cohabitation with a partner (+ 0.29) were associated with higher BMI, whereas smoking (-0.52) and greater sports participation (-0.13) were associated with lower BMI; the smoking association likely reflects, at least partly, weight gain after cessation (smoking prevalence fell from 30% to 20%). Women experienced a stronger BMI increase than men only at the last measurement point (2023/24), and those with tertiary education (-0.02) showed slower BMI increases.

**Conclusions:**

Body weight and weight gain are associated with socioeconomic, behavioral, and psychological factors. Among the Big Five personality traits, conscientiousness showed the strongest association with lower BMI, whereas neuroticism was unexpectedly associated with lower rather than higher BMI and warrants replication with more comprehensive personality measures. Higher educational attainment and more frequent physical activity were associated with lower BMI and, within individuals, with more favorable weight trajectories; given the exploratory design, these associations should not be interpreted causally. The findings highlight the relevance of psychological characteristics for obesity research and prevention.

**Supplementary Information:**

The online version contains supplementary material available at https://doi.org/10.1186/s12889-026-30138-w.

## Background

The prevalence of obesity in Germany is already high and continues to increase. As of 2025, 17.9% of the German population is classified as obese, representing a substantial increase from 16.8% in 2021 and 14.7% in 2009 [1]. Overall, 53.4% of Germans are either overweight or obese, indicating that more than half of the population currently has an unhealthy body weight [2]. Given the strong association between excess weight and adverse health outcomes [3], these trends are particularly concerning in the context of an aging society [4]. Body weight is determined by a wide range of interacting factors. Established frameworks such as the Dahlgren–Whitehead model of the main determinants of health [5] and the Foresight obesity system map [6] place individual lifestyle factors within social and community networks, living and working conditions, and the wider socioeconomic environment. A large body of research has accordingly identified factors associated with overweight and obesity, including sex, educational attainment, partnership status, socioeconomic disadvantage, smoking behavior, dietary habits, and physical activity. For most of these factors, systematic reviews or large cohort studies are available. Reviews document associations of obesity with socioeconomic status [7] and educational attainment [8]. Transitions into marriage or cohabitation have been linked to weight gain [9], and weight gain after smoking cessation is well established [10]. Evidence on alcohol consumption is inconsistent [11], and specific dietary and lifestyle behaviors have been shown to be independently associated with long-term weight gain in large prospective cohorts [12]. However, individual studies typically examine only a limited set of these factors, and results are not always consistent, especially regarding the measures of income and wealth, as some studies report positive, others negative associations with weight. Psychological characteristics have received growing attention in this context, as personality traits are related to health-related behaviors and self-regulation. An individual-participant meta-analysis of nine cohort studies found that higher conscientiousness was associated with a lower risk of developing and maintaining obesity [13]. A further meta-analysis confirmed a negative association of BMI with conscientiousness and a positive association with neuroticism at the domain level, with the associations being driven primarily by narrow traits [14]. Likewise, a systematic review identified neuroticism, impulsivity, and sensitivity to reward as risk factors, and conscientiousness and self-control as protective factors for overweight and obesity [15], and longitudinal analyses of BMI trajectories suggest that impulsivity-related traits predict greater weight gain across the adult life span [16]. However, findings differ depending on the study design and the personality measure used, and much of the evidence is cross-sectional or based on international cohorts. Evidence for Germany is comparatively scarce: to our knowledge, only one study based on the German Socio-Economic Panel has examined personality and obesity longitudinally, using two measurement points and a binary obesity indicator. It found that most associations observed cross-sectionally were no longer present in fixed-effects models [17]. We therefore add to this literature by examining personality traits jointly with socioeconomic and behavioral factors in a German adult cohort followed for 12 years, combining cross-sectional models with fixed-effects models of BMI change.

To address these gaps, we leverage data from a large prospective panel study including more than eight thousand participants to improve understanding of the factors associated with body weight and weight change over time. Rather than testing hypotheses on individual factors, this study takes an exploratory, descriptive approach and provides a systematic overview of socioeconomic, behavioral, and psychological factors associated with BMI and BMI change among adults in Germany, similar to analyses conducted for other countries such as Switzerland [18].

## Methods

### The National Educational Panel Study (NEPS)

The National Educational Panel Study (NEPS) is the largest German longitudinal study investigating the role of education across the life course [19].^1^ Designed as a multi-cohort sequential study, the NEPS has collected prospective panel data from a diverse range of cohorts since 2010/11. Although its primary focus is on educational processes and outcomes, the NEPS also gathers extensive information on labor market participation, sociodemographic characteristics, health indicators, contextual factors, and a broad range of psychological measures. Taken together, these data provide a unique opportunity to investigate the factors associated with changes in body weight over time.

For the present study, we use data from the adult cohort (NEPS-SC6), which comprises adults born between 1944 and 1986 and living in Germany [20, 21]. Participants were drawn as a probability sample from local population registers (*Einwohnermeldeamtsstichprobe*), covering residents of Germany irrespective of nationality or German language proficiency [22]. The cohort combines respondents originally surveyed in 2007/08 as part of the “Working and Learning in a Changing World” (ALWA) study, together with subsequent NEPS enhancement and refreshment samples [22]. Data collection has been carried out via computer-assisted telephone and personal interviews (CATI/CAPI) since 2009/10. Wave 4 was selected as the baseline (t1) because it was the first wave in which body weight was assessed (2011/12). Follow-up assessments of body weight were conducted in waves 7 (t2; 2014/15), 10 (t3; 2017/18), 12 (t4; 2019/20), 13 (t5; 2020/21), and 16 (t6; 2023/24), resulting in a longitudinal panel with six measurement occasions spanning approximately 12 years.

### Socio-economic data

Age was measured in years at baseline (wave 4). Highest educational attainment was classified into three categories: (1) below higher education entrance qualification (*Abitur*), (2) higher education entrance qualification (*Abitur* or equivalent), and (3) any tertiary educational qualification. Sex was measured as male or female. Place of residence was classified as either East or West Germany. Missing information on place of residence was replaced by the nearest observed value of the same person. Because the fixed-effects estimate for living in East Germany is identified only by individuals who moved between East and West Germany, we note that this applies to 141 individuals (1.7%) of the analytic sample. Migration background was measured as a binary indicator and was defined as either the respondent or at least one parent having been born outside Germany. To this end, the generation status provided by the NEPS was collapsed: first to 2.75th generation (respondent or at least one parent born abroad) = 1; no migration background or third generation and later (only grandparents born abroad) = 0. Employment status was measured in each survey wave as a binary indicator of whether an employment spell was ongoing at the interview date. Unemployment was measured as a binary indicator of whether an unemployment spell occurred in the 12 months before the interview, derived from the NEPS life-course data. Respondents who were neither employed nor recently unemployed (e.g., retired persons or homemakers) thus form the reference group. Living with a partner or spouse and living with children (biological, adopted, or stepchildren) were also measured as binary variables in each wave. Occupational status was assessed using the International Socio-Economic Index of Occupational Status (ISEI) [23]. In each wave, we used the highest ISEI of all employment spells reported in that wave, including jobs that had ended since the previous interview. Because occupation is recorded at the start of an employment spell, ISEI values were carried forward within continuing spells; remaining missing values were imputed. Post-tax household income (log-transformed) and household size were measured in each survey wave. Social origin was assessed using five indicators: father’s and mother’s highest school-leaving qualification, father’s and mother’s occupational status (ISEI-08), and whether the father held a management position. All parental information is time-constant and was taken from the first wave in which it was available. These indicators were combined into a latent factor using a one-factor confirmatory factor analysis estimated by maximum likelihood with missing values; the predicted factor scores were standardized (z-score). Higher values indicate a more advantaged socioeconomic background.

### Body mass index, health behaviors, and health status

Body weight and height were self-reported in each survey wave and used to calculate body mass index (BMI; weight in kilograms divided by height in meters squared, kg/m²). Reporting bias is common for these measurements, which we also discuss in the limitations. Based on the World Health Organization (WHO) classification, normal weight was defined as a BMI of 18.5 to < 25.0 kg/m², overweight as a BMI of 25.0 to < 30.0 kg/m², and obesity as a BMI of ≥ 30.0 kg/m² [24]. Smoking status was measured as a binary variable in each survey wave. The frequency of alcohol consumption was assessed in each wave; for time points 1 and 2, information from point 3 was used for imputation. Similarly, the frequency of sports participation was measured at each time point, with values for measurement points 1 and 2 imputed from time point 3. Mental and physical health were assessed in NEPS wave 11 using the established German Socio-Economic Panel (SOEP) health instruments (SF-12) [25, 26].

### Psychological measurements

Personality traits were assessed in wave 4 (2011/12) using the Big Five Inventory, covering the five broad personality dimensions of Openness to Experience, Conscientiousness, Extraversion, Agreeableness, and Neuroticism (BFI-10) [27]. Willingness to take risks was measured in wave 8 (2015/16). A composite risk-taking score was constructed using principal component analysis based on four domains: financial, leisure-related, career-related, and general risk-taking [28]. The resulting scale demonstrated acceptable internal consistency (Cronbach’s α = 0.74). Global self-worth was assessed in wave 3 (2010/11) using an established self-esteem instrument [29]. Life satisfaction was measured in each survey wave using the Cantril ladder, with scores ranging from 0 to 10, where higher values indicate greater satisfaction.

### Statistical analysis

Participants were included if they were between 28 and 55 years of age at baseline. This age range was chosen to ensure that respondents had typically completed their initial educational qualifications and were likely to be active in the labor market. Participants who never reported their body weight (74 individuals) or who reported being underweight (BMI < 18.5 kg/m²) in any survey wave were excluded from the analyses (146 individuals). We excluded these individuals entirely rather than only their underweight observations because underweight in adulthood frequently reflects illness, eating disorders, or other specific conditions whose weight dynamics differ from the processes studied here. We acknowledge that this conditions the sample on the outcome; however, given the small number of affected individuals (1.7% of the age-eligible sample), this choice is unlikely to affect the results. The BMI variable was winsorized at a level of 45 (values above 45 were set to 45 before imputation) to avoid distortions of the linear models due to extreme outliers (affecting 0.6% of all data points). Of the 182 affected values, 130 lay between 45 and 50 and only two exceeded 60. The procedure lowers the mean of the observed BMI values by 0.03 points. The binary overweight and obesity indicators are unaffected by this, as values above 45 exceed both thresholds. One individual without any information on place of residence was also excluded. After applying these inclusion and exclusion criteria, the final analytical sample comprised 8,209 individuals.

**Fig. 1:**
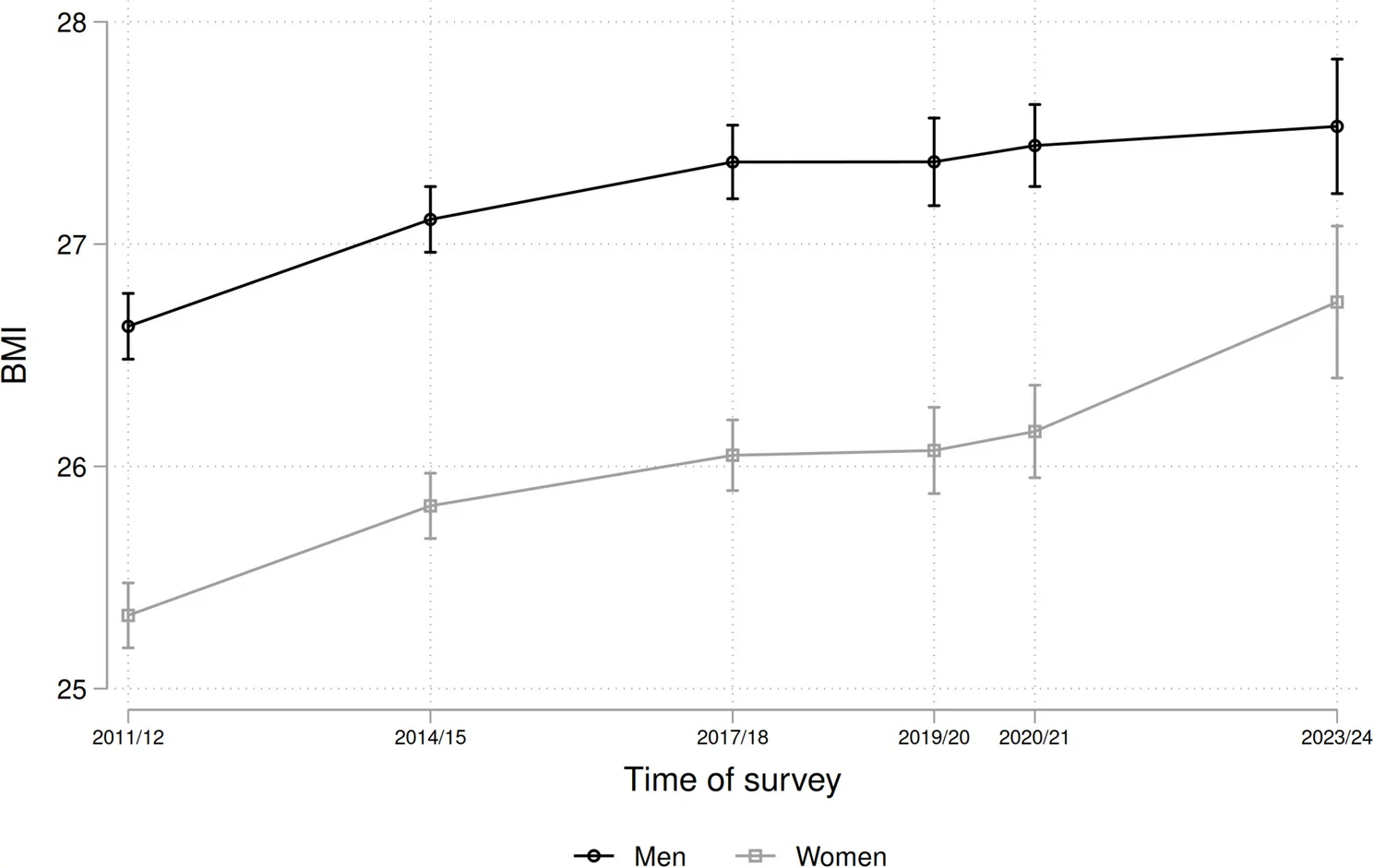
Mean BMI over time, by sex. Source: NEPS SC6, imputed data (*N*=8,209; M=50). 95% confidence intervals included. Graph is purely descriptive as no controls are included. Figure A1 in the online appendix shows the same graph based on observed cases only

**Fig. 2:**
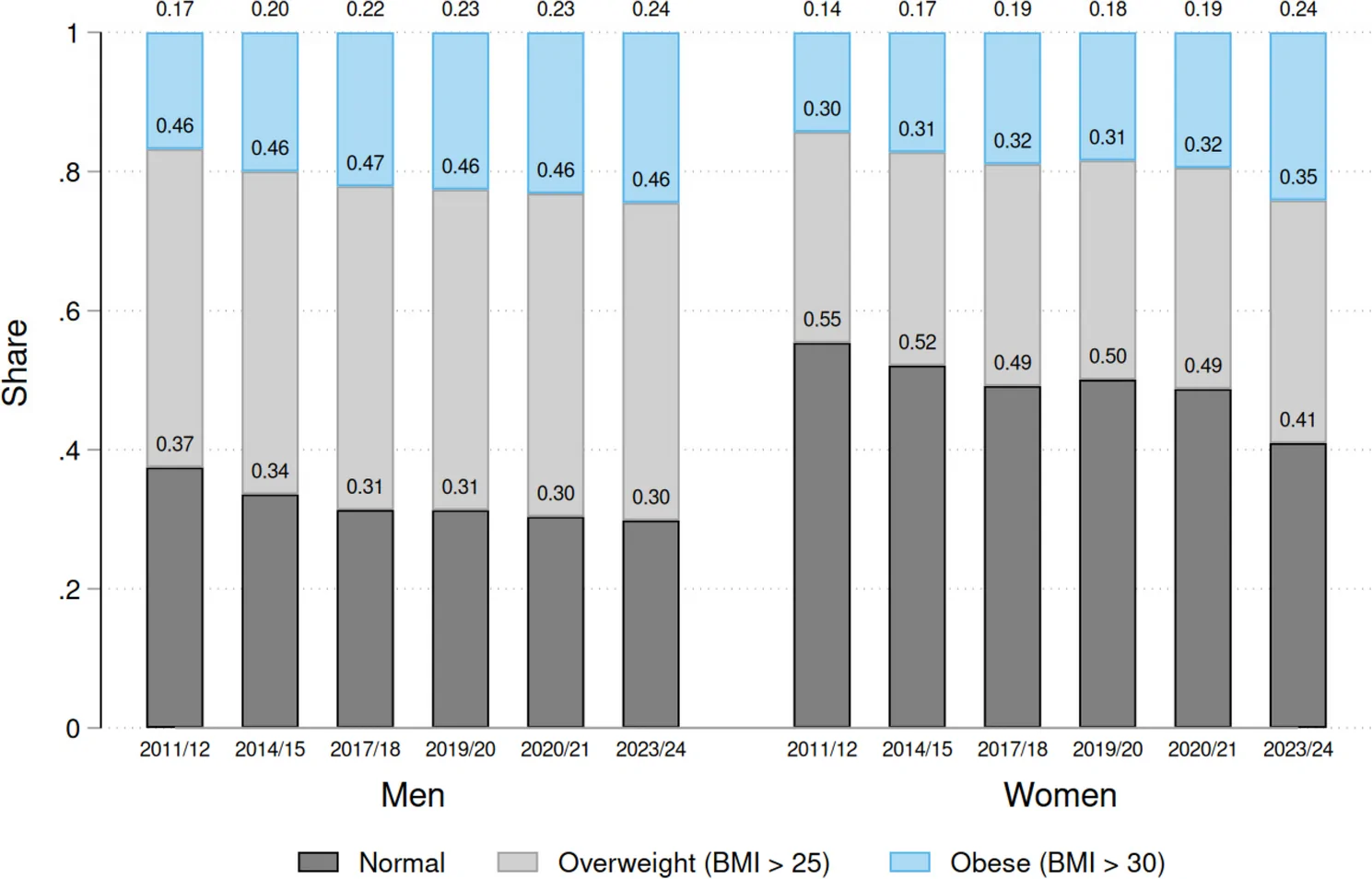
BMI categories (WHO definition) for each survey point, by sex. Source: NEPS SC6, imputed data (*N*=8,209; M=50). Graph is purely descriptive as no controls are included. Figure A2 in the online appendix shows the same graph based on observed cases only

All analyses were conducted using Stata 18.0 [30]. Missing data due to item- and unit-nonresponse were addressed using multiple imputation by chained equations (MICE), generating 50 imputed datasets [31]. Imputation was performed in wide format (one variable per measurement point) and separately for women and men, so that all associations in the imputation model could differ by sex, in line with the sex-specific trajectories and sex-stratified models analysed below. The imputation model additionally included auxiliary variables that are not part of the analysis models (body height, interest in mathematics and in German, and an indicator of everyday physical activity such as walking or cycling). BMI was observed for 6,026 (73%), 6,287 (77%), 5,317 (65%), 4,825 (59%), 4,520 (55%), and 3,264 (40%) of the 8,209 individuals at measurement points 1 to 6, respectively; the remaining values were imputed. Descriptive statistics and Figs. 1 and 2 based on observed cases only are provided in the online appendix (Table A7, Figures A1 and A2). We did not apply NEPS’s nonresponse-adjusted weights. These are calibrated on sociodemographic variables with known population benchmarks and would not correct for selective attrition on the psychological and health-related variables central to this study; their combination with multiply imputed data would additionally require a weighted imputation procedure beyond the scope of this analysis (see Limitations). Because alcohol consumption and sports participation at the first two measurement points were imputed from the third measurement point, we additionally estimated the fixed-effects models restricted to measurement points 3 to 6 as a sensitivity check (Table A3). Because mental and physical health (wave 11) and risk-taking (wave 8) were measured after the baseline and may be consequences rather than determinants of body weight, we additionally estimated the baseline models without these variables (Table A2). Descriptive statistics are reported as means, standard deviations, medians, and minimum and maximum values. A complete correlation table is provided in the online appendix. Variables without a readily interpretable metric were standardized (z-scores) to facilitate comparison of effect sizes across correlates. To examine associations with BMI at baseline, ordinary least squares (OLS) regression models with robust standard errors were estimated; for the binary outcome variable, this is a linear probability model [32]. As a measure of explanatory power, we report R² for the baseline models. Associations with changes in BMI over time were analyzed using fixed-effects panel regression models with robust standard errors. Fixed-effects models account for all time-invariant observed and unobserved individual characteristics and are therefore well suited for analyzing longitudinal observational data [33]. For time-invariant variables, such as sex, interactions with time since baseline were included to assess whether trajectories of BMI differed across groups over the observation period. Age at baseline was centred at 44 years, so that the coefficient of time since baseline refers to a 44-year-old man with low education, no migration background, and average values on all standardized variables. Time since baseline was modelled linearly, which assumes that BMI and group differences in BMI change at a constant rate over the observation period. To assess this assumption, we re-estimated the fixed-effects models with dummy variables for each measurement point instead of the linear time variable (Table A8). Statistical significance was defined as *p* < 0.05. Since a large number of correlates is included in the models, we regard the analyses as exploratory since no distinct and directed hypotheses are presented for all independent variables.

## Results

### Descriptive results

Descriptive statistics for all baseline variables are presented in Table 1 and for time-variant variables in the online appendix because of the substantial length of the corresponding tables (Table A1 and, based on observed cases only, in Table A7). Question text and item wordings are provided in the appendix (Table A6).

**Table 1:** Sample characteristics at baseline

	Mean	SD	Median	Min	Max
Female respondent	0.51	0.50	1	0	1
Age at baseline	44.2	7.59	46	28	55
Migration background	0.18	0.38	0	0	1
Education level					
Low	0.51	0.50	1	0	1
Higher education eligibility	0.18	0.39	0	0	1
Tertiary	0.31	0.46	0	0	1
Social origin	0.075	1.02	-0.26	-1.65	2.85
B5: Extraversion	3.39	0.85	3.50	1	5
B5: Agreeableness	3.56	0.54	3.67	1.33	5
B5: Conscientiousness	4.03	0.64	4	1	5
B5: Neuroticism	2.61	0.71	2.50	1	5
B5: Openness	3.44	0.84	3.50	1	5
Self-worth	43.1	4.62	44	19	50
Risk taking	0.0047	1.48	0.043	-5.01	4.93
Mental health score	50.4	9.27	51.8	8.62	75.6
Physical health score	51.8	8.56	53.6	12.6	73.0
BMI (t1)^#^	26.0	4.39	25.3	18.5	45.0
Frequency doing sports (t1) ^#^	2.99	1.19	3.00	1.00	5.00
Smoking (t1) ^#^	0.30	0.46	0	0	1
Observations	8209				

Source: NEPS SC6, imputed data (N=8,209; M=50)

^#^time-variant variable at t1

Key sample characteristics are as follows: 51% of respondents are female, 18% report a migration background, and the mean age at baseline is 44.2 years. Furthermore, 20% of participants reside in East Germany at baseline, and 31% have attained a tertiary educational qualification. Pairwise correlations (Pearson’s r) at baseline (see online appendix, Excel file) among the 27 predictors (with education entered as two dummy variables, as in the models) were mostly small, with a median absolute correlation of 0.05. Only two of the 351 pairs exceeded |r| = 0.5: household size and living with children (*r* = 0.70), and tertiary education and occupational status (ISEI; *r* = 0.57). All other correlations were below 0.45. Correlations of the predictors with BMI were at most |r| = 0.31 (physical health), followed by social origin, sex, and sports participation (each about − 0.15). Variance inflation factors were low (maximum 2.5, for household size; mean 1.4), indicating that collinearity is unlikely to affect the estimates materially.

Figure 1 illustrates the mean BMI over time, separately for men and women. Several observations can be made. First, the average BMI exceeds 25 kg/m² in all survey waves, indicating that the average respondent falls within the overweight category throughout the observation period. Second, mean BMI is consistently higher among men than among women, as evidenced by the higher trajectory across all measurement occasions. Third, BMI increases over time for both sexes, reflecting a general trend toward weight gain during the study period. Fourth, the increase in BMI appears to become steeper among women after 2020, whereas the trajectory for men remains comparatively stable. Men have a higher mean BMI than women at all survey waves; however, the difference between the two groups has become much smaller, from 1.30 BMI points in 2011/12 to 0.79 points in 2023/24. Since a substantial share of BMI values at the later measurement points is imputed, Figures A1 and A2 in the online appendix present the same descriptive results based on observed cases only. The general increase in BMI is similar in the observed data (about 1.2 vs. 1.1 BMI points over twelve years), whereas the convergence between men and women in 2023/24 is less pronounced (difference of 1.04 BMI points). As the measurement at t5 (2020/21) falls within the COVID-19 pandemic, this pattern may partly reflect pandemic-related changes in daily routines and physical activity; the present data and the scope of this paper do not allow us to examine this further.

To better understand how these arithmetic means translate to WHO categories, the following figure summarizes the results for men and women by survey wave (Fig. 2).

Substantial changes in BMI categories can be observed over the study period. Among men, the proportion with a normal BMI decreased from approximately 38% at baseline to 30% after 12 years of follow-up. Among women, the corresponding proportion declined from approximately 55% to 43%. At the same time, the prevalence of obesity increased markedly in both groups. Among men, the proportion classified as obese rose from 17% at baseline to 24% at the final observation. Among women, the prevalence of obesity increased from 15% to 22% over the same period. In the observed data, the increase in obesity is very similar for both sexes (men: from 16% to 24%; women: from 14% to 21%; Figure A2).

### Multivariate results

Table 2 reports the regression results for both outcome variables: BMI as a continuous measure (Model 1) and overweight/obesity status (Model 2), defined as a BMI of at least 25 kg/m². Higher household income, higher occupational status (ISEI), and a tertiary qualification are associated with lower BMI; occupational status and a tertiary qualification are also associated with a lower probability of being overweight or obese, whereas household income is not significantly related to this outcome. Compared with individuals with low educational attainment, respondents with a tertiary qualification have an approximately five-percentage-point lower probability of being overweight or obese and a BMI that is, on average, 0.41 points lower. Women exhibit lower BMI values than men, and younger individuals have lower BMI values than older individuals.

Several health-related behaviors are also associated with BMI. Smokers report lower BMI values than non-smokers, while more frequent alcohol consumption is associated with lower BMI. In addition, individuals who engage in sports more frequently tend to have lower BMI values.

A number of psychological characteristics are statistically significantly associated with BMI. Higher levels of conscientiousness and neuroticism are associated with lower BMI, whereas higher levels of extraversion are associated with higher BMI. Conscientiousness shows the strongest association among the personality traits (-0.64 BMI points per standard deviation), followed by neuroticism (-0.27) and extraversion (+ 0.23); agreeableness and openness are not significantly related to BMI. Better physical health is strongly associated with lower BMI; however, the direction of causality cannot be determined from the available data. Furthermore, individuals with a greater willingness to take risks tend to report lower BMI values.

Larger households are associated with higher BMI (0.121) and a higher probability of being overweight or obese (0.015), and respondents living in East Germany have a higher probability of being overweight or obese (0.039), although not a significantly higher BMI. Higher agreeableness is associated with a slightly lower probability of being overweight or obese (-0.014) but not significantly with BMI. The remaining variables (employment, unemployment, living with a partner or with children, migration background, higher education eligibility, openness, self-worth, mental health, and life satisfaction) are not significantly associated with BMI or overweight/obesity status. Finally, the explanatory power of the models, as indicated by the R² values, remains modest. Despite the inclusion of a large number of correlates, less than 20% of the variation in BMI is explained. When risk taking and mental and physical health are omitted (Table A2), most estimates remain stable in direction and significance, while socioeconomic gradients become larger, for example for tertiary education (-0.618 vs. -0.413) and household income (-0.226 vs. -0.155); life satisfaction then shows a significant negative association with BMI (-0.139).

**Table 2:** Regression coefficients at baseline (linear models; 2011/12)

	M1	M2
	BMI (continuous)	Being overweight/obese
Log. HH income°	-0.155^*^	-0.007
	[-0.292,-0.017]	[-0.024,0.009]
Employed	-0.039	0.005
	[-0.421,0.343]	[-0.036,0.046]
Unemployment episode reported	0.330	0.011
	[-0.078,0.739]	[-0.030,0.053]
ISEI°	-0.148^*^	-0.021^**^
	[-0.274,-0.022]	[-0.036,-0.006]
Living together with partner	0.318	0.036
	[-0.048,0.685]	[-0.004,0.077]
Living together with children	-0.116	-0.005
	[-0.400,0.168]	[-0.038,0.028]
Number of people in the HH	0.121^*^	0.015^*^
	[0.007,0.236]	[0.001,0.029]
Living in East Germany	0.202	0.039^**^
	[-0.031,0.436]	[0.011,0.067]
Life satisfaction°	-0.014	0.001
	[-0.130,0.101]	[-0.012,0.014]
Smoking	-0.911^***^	-0.072^***^
	[-1.124,-0.698]	[-0.097,-0.046]
Frequency alcohol consumption°	-0.403^***^	-0.030^***^
	[-0.510,-0.296]	[-0.044,-0.017]
Frequency doing sports°	-0.383^***^	-0.038^***^
	[-0.492,-0.274]	[-0.051,-0.025]
Female respondent	-1.419^***^	-0.182^***^
	[-1.627,-1.211]	[-0.207,-0.157]
Age at baseline	0.036^***^	0.006^***^
	[0.022,0.050]	[0.004,0.007]
Migration background	0.116	0.018
	[-0.127,0.359]	[-0.010,0.047]
Low education	Ref.	Ref.
HEE (Abitur)	-0.245	-0.029
	[-0.505,0.016]	[-0.061,0.003]
Tertiary	-0.413^**^	-0.048^**^
	[-0.687,-0.140]	[-0.081,-0.015]
Social origin°	-0.260^***^	-0.029^***^
	[-0.367,-0.154]	[-0.042,-0.016]
B5: Extraversion°	0.227^***^	0.023^***^
	[0.114,0.340]	[0.010,0.036]
B5: Agreeableness°	-0.099	-0.014^*^
	[-0.199,0.000]	[-0.026,-0.002]
B5: Conscientiousness°	-0.640^***^	-0.053^***^
	[-0.747,-0.533]	[-0.065,-0.041]
B5: Neuroticism°	-0.269^***^	-0.020^**^
	[-0.382,-0.155]	[-0.033,-0.007]
B5: Openness°	0.053	-0.002
	[-0.049,0.155]	[-0.014,0.010]
Self-worth°	-0.013	0.001
	[-0.147,0.120]	[-0.014,0.016]
Risk taking°	-0.160^**^	-0.011
	[-0.273,-0.047]	[-0.024,0.002]
Mental health score°	0.004	0.007
	[-0.119,0.127]	[-0.006,0.021]
Physical health score°	-1.105^***^	-0.087^***^
	[-1.222,-0.989]	[-0.099,-0.074]
Constant	24.922^***^	0.330^***^
	[24.074,25.769]	[0.228,0.433]
R² (%)	18.27	13.18
Observations	8209	8209

Source: NEPS SC6, imputed data (*N*=8,209; M=50). 95% confidence intervals included, based on robust standard errors

° Variable is z-standardized

* *p* < 0.05, ** *p* < 0.01, *** *p* < 0.001 Physical and mental health refer to wave 11, risk taking to wave 8

We next turn to the fixed-effects models examining changes in BMI over time. The results are presented in Table 3. In this model, time-invariant variables are absorbed by individual fixed-effects and the reported coefficients reflect differences in trajectory slopes over time. The first part of the table reports coefficients for time-varying variables and therefore reflects within-person associations over the observation period. For the reference person (a 44-year-old man with low education; see Methods), BMI increases by 0.076 points per year since baseline. Increases in household income are associated with higher BMI, transitions into unemployment show no significant association with BMI, whereas being employed is associated with slightly lower BMI (-0.13 points). Individuals who begin living with a spouse or partner also experience minor increases in BMI. In contrast, increasing participation in sports is associated with reductions in BMI over time. Regarding smoking, the within-person coefficient indicates that BMI is on average 0.52 points lower at measurement points at which respondents smoked than at points at which the same respondents did not smoke. As smoking prevalence declined from 30% to 20% over the observation period in the observed data (see Table A7), this estimate likely reflects, at least partly, weight gain after smoking cessation, although the fixed-effects estimate itself does not distinguish cessation from initiation. Restricting the fixed-effects models to measurement points 3 to 6 (Table A3) leaves the association with sports participation virtually unchanged (-0.142 vs. -0.129) and yields a smaller but still significant smoking coefficient (-0.356 vs. -0.515), whereas the associations with household income and living with a partner remain significant and of similar size (0.085 and 0.351); the association with employment is no longer significant. The linear time trend is smaller and not significant in this specification (0.033), because the largest increase occurs between the first two measurement points, which are excluded here. The female × time interaction, in contrast, is larger in this specification (0.089 vs. 0.029 in Table 3): without the first two measurement points, the increase among women at the last measurement point (see Table A8) dominates the estimated difference in slopes.

The second part of Table 3 reports interaction effects between time and time-invariant characteristics. These coefficients indicate whether BMI trajectories differ across population subgroups. Women experience somewhat larger increases in BMI over time than men (0.029 points per year), but this difference is not statistically significant. Furthermore, younger individuals at baseline show steeper increases; and respondents with a tertiary qualification exhibit slower increases in BMI over the observation period (-0.023), as indicated by the negative interaction coefficient; the interaction for higher education eligibility is not significant. In contrast, no statistically significant differences in BMI trajectories are observed for the remaining time-invariant characteristics included in the analysis, with the exception of mental health (better mental health is associated with a slower increase). None of the Big Five traits shows a significant interaction with the linear time trend.

Because the linear specification imposes a constant rate of change, we re-estimated the fixed-effects models with dummy variables for each measurement point (Table A8). The coefficients of the time-varying variables are virtually unchanged (e.g., household income: 0.102; living with a partner: 0.276; smoking: -0.515; sports participation: -0.133), and the gap by tertiary education widens gradually over time, as assumed by the linear model. The overall time trend, however, is not linear: for a 44-year-old man with low education, BMI increases by 0.52 points in the first three years and by 0.94 points over the full observation period, so that about half of the increase occurs between 2011/12 and 2014/15. The linear coefficient thus reflects the overall increase over twelve years (0.91 points) well, but not its shape. Among the personality traits, only conscientiousness shows small negative interactions at several measurement points (about − 0.08 to -0.09 BMI points between 2014/15 and 2020/21, but not at the last measurement point); given the large number of interaction coefficients, we regard this as exploratory. Likewise, the steeper increase among women is not a gradual divergence: the trajectories of women and men run parallel until 2020/21 and diverge only at the last measurement point in 2023/24 (0.51 BMI points). As BMI values at this measurement point are imputed to a larger extent, and the convergence between men and women is less pronounced in the observed data (Figure A1), this result should be interpreted with caution.

**Table 3:** FE regression results

	M3	M4
	BMI (continuous)	Being overweight/obese
Time-variant variables		
Years since baseline	0.076^***^	0.007^***^
	[0.055,0.098]	[0.004,0.009]
Log. HH income°	0.095^**^	0.008
	[0.032,0.159]	[-0.002,0.018]
Employed	-0.129^*^	-0.007
	[-0.236,-0.022]	[-0.024,0.010]
Unemployment episode reported	0.074	0.008
	[-0.047,0.194]	[-0.009,0.025]
ISEI°	-0.051	-0.008
	[-0.149,0.047]	[-0.021,0.005]
Living together with partner	0.288^**^	0.031^*^
	[0.094,0.482]	[0.006,0.057]
Living together with children	-0.014	0.002
	[-0.131,0.102]	[-0.016,0.020]
Number of people in the HH	-0.019	-0.001
	[-0.075,0.037]	[-0.009,0.008]
Living in East Germany	-0.161	-0.044
	[-0.498,0.176]	[-0.102,0.014]
Life satisfaction°	0.036^*^	0.002
	[0.005,0.067]	[-0.003,0.007]
Smoking	-0.515^***^	-0.046^***^
	[-0.638,-0.393]	[-0.065,-0.027]
Frequency alcohol consumption°	0.084^*^	0.007
	[0.015,0.153]	[-0.002,0.017]
Frequency doing sports°	-0.129^***^	-0.010^**^
	[-0.178,-0.081]	[-0.016,-0.003]
Time-constant variables		
Female respondent^#^	0.029	0.002
	[-0.005,0.063]	[-0.002,0.005]
Age at baseline (centred at 44)^#^	-0.004^***^	-0.000^***^
	[-0.005,-0.003]	[-0.001,-0.000]
Migration background^#^	0.004	-0.000
	[-0.015,0.024]	[-0.003,0.003]
Low education	Ref.	Ref.
HEE (Abitur) ^#^	-0.005	0.000
	[-0.024,0.013]	[-0.003,0.003]
Tertiary^#^	-0.023^**^	-0.001
	[-0.039,-0.007]	[-0.003,0.002]
Social origin°^#^	-0.002	-0.000
	[-0.010,0.005]	[-0.001,0.001]
B5: Extraversion°^#^	0.004	0.001
	[-0.004,0.012]	[-0.000,0.002]
B5: Agreeableness°^#^	0.002	0.001
	[-0.006,0.010]	[-0.000,0.002]
B5: Conscientiousness°^#^	-0.006	0.000
	[-0.013,0.001]	[-0.001,0.001]
B5: Neuroticism°^#^	0.001	0.000
	[-0.007,0.008]	[-0.001,0.001]
B5: Openness°^#^	0.002	-0.000
	[-0.005,0.009]	[-0.001,0.001]
Self-worth°^#^	-0.001	0.001
	[-0.009,0.008]	[-0.001,0.002]
Risk taking°^#^	0.000	0.001
	[-0.008,0.008]	[-0.001,0.002]
Mental health score°^#^	-0.012^**^	-0.002^**^
	[-0.020,-0.004]	[-0.003,-0.001]
Physical health score°^#^	-0.004	0.001
	[-0.013,0.004]	[-0.000,0.002]
Constant	26.176^***^	0.540^***^
	[25.934,26.419]	[0.503,0.577]
Individuals	8209	8209
Observations	49254	49254

Source: NEPS SC6, imputed data (N=8,209; M=50). 95% confidence intervals included, based on robust standard errors. Living in East Germany is identified by the 141 individuals (1.7%) who moved between East and West Germany. Age at baseline centred at 44 years; the time coefficient refers to a 44-year-old man with low education, no migration background, and average values on all standardized variables

° Variable is z-standardized

* *p* < 0.05, ** *p* < 0.01, *** *p* < 0.001

^#^ Interacted with time variable

Finally, both main models are re-estimated when stratifying for sex to increase insight (Appendix, tables A4 and A5). Results showed that most associations were similar for women and men. In the baseline models, smoking, alcohol consumption, sports participation, social origin, conscientiousness, neuroticism, and physical health were associated with lower BMI in both groups. Some estimates differed descriptively (men vs. women): household income (0.057 vs. -0.368), occupational status (0.033 vs. -0.386), and conscientiousness (-0.472 vs. -0.812) were more strongly associated with lower BMI among women, whereas extraversion was associated with higher BMI among men only (0.323 vs. 0.151). Tertiary education was significantly associated with lower BMI among men (-0.538) but not among women (-0.399); as the point estimates are similar in size and the confidence intervals overlap widely, this should not be read as a sex difference. Openness (0.181) and risk taking (-0.233) were significantly associated with BMI among women only. In the fixed-effects models, smoking and sports participation were associated with lower BMI in both groups, and BMI increased faster among women (0.07 vs. 0.11 points per year; the difference is not statistically significant, see Table 3). Increases in household income (0.049 vs. 0.130) and living with a partner (0.159 vs. 0.358) were associated with higher BMI mainly among women, and alcohol consumption mainly among men (0.142 vs. 0.026). Again, coefficients are to be interpreted descriptively as it was not the main goal of the study to investigate sex differentials.

## Discussion

Given the increasing prevalence of overweight and obesity in Germany [34], with substantial consequences for population health and healthcare expenditure, the issue of overweight and obesity is clearly relevant and important to study in detail. The present study contributes new evidence by drawing on a large prospective German cohort with a broad range of sociodemographic, behavioral, and psychological measures. Its most noteworthy finding concerns personality: conscientiousness and, unexpectedly, also neuroticism were associated with lower BMI, whereas extraversion was associated with higher BMI. The findings indicate that several socioeconomic factors are associated with body weight. Individuals with higher incomes and higher educational attainment exhibit lower BMI values, and those with higher educational attainment or occupational status also lower probabilities of being overweight or obese. These patterns are consistent with previous research suggesting that socioeconomic resources are closely linked to health-related behaviors and health outcomes [35–37]. One possible explanation is that individuals with higher educational attainment possess greater health literacy and are therefore better able to adopt and maintain healthy lifestyles [38].

Several behavioral factors are also associated with BMI. More frequent participation in sports is consistently associated with lower body weight, highlighting the importance of physical activity for weight management. The negative cross-sectional association between smoking and BMI likely reflects a combination of factors rather than a single mechanism. Nicotine is known to suppress appetite and increase resting energy expenditure [39], which may partly explain lower BMI among current smokers; at the same time, smoking and BMI may share common confounders, such as socioeconomic status and health-related self-control. The negative association should not be read as a beneficial effect of smoking: smoking is a major risk factor for cardiovascular disease, cancer, and other non-communicable diseases [40], and, as noted above, the within-person coefficient likely reflects, at least partly, cessation-related weight gain rather than a causal effect of smoking itself.

The negative cross-sectional association between alcohol consumption and BMI should not be interpreted as a protective effect of alcohol either. Alcohol consumption is itself a risk factor for cardiovascular disease, several cancers, and other non-communicable diseases [41], and its negative association with BMI here is likely to reflect confounding rather than any protective mechanism. Turning to psychological characteristics, the role of personality deserves particular attention. Among the Big Five personality dimensions, conscientiousness shows the strongest association with lower BMI, whereas extraversion is associated with higher BMI. The strong association between conscientiousness and lower BMI is plausible given the defining features of the trait. Conscientious individuals tend to show greater impulse control, are more likely to plan ahead and to delay gratification, and behave more consistently in pursuit of long-term goals. These characteristics may facilitate healthier everyday routines, such as planning meals, resisting energy-dense food in tempting situations, and exercising regularly, and meta-analytic evidence shows that conscientiousness is positively related to beneficial and negatively related to risky health-related behaviors [42]. As these mechanisms were not measured directly, they should be understood as plausible interpretations rather than tested pathways. Notably, conscientiousness was not related to BMI trajectories in the fixed-effects models, suggesting that the trait is linked more to differences in BMI levels than to the rate of weight gain (the linear interaction is not significant; the specification with measurement-point dummies in Table A8 shows small negative interactions at the intermediate waves).

The negative association between neuroticism and BMI was unexpected, as meta-analytic evidence points to a positive domain-level association [14], and neuroticism, particularly its impulsivity-related aspects, has been identified as a risk factor for higher adiposity and weight gain [15, 16]. Several explanations should be considered. First, our model adjusts for characteristics that are closely related to neuroticism, namely mental health, self-worth, and life satisfaction, as well as for smoking, alcohol consumption, and risk taking. Conditioning on these variables may have removed the part of neuroticism that is linked to higher BMI, leaving a residual component with a different sign. However, the negative association persists when mental and physical health and risk taking are omitted (Table A2: -0.217), so this adjustment cannot be the only explanation. A previous analysis of German panel data likewise found a positive unadjusted but no adjusted association [17]. Second, personality was measured with the 10-item short form of the Big Five Inventory [27], which captures each trait with only two items. This instrument is acceptable for group-level research, but it represents neuroticism mainly through nervousness and stress reactivity rather than impulsivity-related facets, and associations with BMI appear to be driven by narrow traits rather than broad domains [14]. Measurement error may also attenuate the coefficient (biased towards zero) and distort the estimates of correlated covariates. Third, associations may differ across subgroups [14], which we did not test beyond the sex-stratified models. Finally, neuroticism was unrelated to BMI trajectories in the fixed-effects models, so the finding concerns BMI levels only. It should therefore be interpreted with caution and replicated using more comprehensive personality measures. The explanatory power of the baseline models was modest (R² = 18.3%). This is plausible, since body weight is also shaped by factors not measured here, such as diet, genetic predisposition, and the food and built environment [6].

The fixed-effects analyses provide additional insights into factors associated with within-individual changes in BMI over time. Although these models cannot establish causality, they account for all stable observed and unobserved individual characteristics and therefore provide a more stringent test of longitudinal associations. Increases in household income are associated with increases in BMI, whereas being employed is associated with slightly lower BMI and unemployment shows no significant association. While the mechanisms underlying these relationships remain unclear, the findings suggest that changes in economic circumstances may be linked to changes in body weight. The within-person association between income and BMI does not simply reflect general aging, since the fixed-effects models already account for the linear increase in BMI over time (via years since baseline). However, income and employment also tend to change with age over the life course, for example due to career progression, so a residual age-related component of these associations cannot be fully excluded. In contrast, increasing participation in sports is associated with reductions in BMI over time. The negative within-person smoking coefficient, on the other hand, should not be read as a beneficial effect of smoking. Given the strong decline in smoking prevalence, it likely reflects, at least partly, the weight gain that typically follows smoking cessation [10]. Among the time-invariant characteristics, women exhibit greater increases in BMI over time than men, although this difference is not statistically significant in the linear specification and emerges only at the last measurement point (2023/24), which partly rests on imputed values. Furthermore, those with a tertiary qualification experience slower increases in BMI over the observation period, although this interaction is not significant when the first two measurement points are excluded (Table A3). Occupational status and social origin were not significantly associated with within-person BMI change.

In the baseline models including mental and physical health, life satisfaction is not significantly associated with BMI. This contrasts with cross-sectional evidence from the German Socio-Economic Panel, where lower life satisfaction is associated with higher BMI [43]. In our data, the association becomes significant once mental and physical health and risk taking are excluded from the model (Table A2), suggesting that part of the cross-sectional relationship between life satisfaction and BMI operates through these health-related variables. In the fixed-effects models, within-person changes in life satisfaction are only weakly associated with BMI change (0.04 BMI points per standard deviation; not significant for overweight/obesity), in line with the more general attenuation of associations observed once time-invariant confounding is addressed. This finding is consistent with previous research from Germany [44]. However, the absence of a statistical association should not be interpreted as evidence that overweight or obesity have no psychological consequences, as well-being may be influenced by a wide range of factors that are not captured by the present analyses.

### Strengths and limitations

A major strength of this study is the use of the National Educational Panel Study (NEPS), which provides prospective longitudinal data for a large sample of adults over an extended observation period. The rich set of available variables allows for a comprehensive investigation of sociodemographic, behavioral, and psychological factors associated with body weight and weight change. In particular, the fixed-effects models represent an important methodological strength, as they exploit within-individual variation over time and account for all time-invariant observed and unobserved characteristics, thereby reducing the risk of bias from stable confounding factors.

Nevertheless, several limitations should be acknowledged. First, participation in the NEPS is voluntary, and panel attrition is documented and mostly unavoidable in long-running panel studies. Wave-specific participation rates among eligible panel members ranged from 75% to 88% across the measurement points used here (excluding the initial wave, which includes a refreshment sample) [45]. Of the original wave 4 participants, about 22% remained in the panel by wave 16. In the age-restricted analytic sample of 8,209 individuals, the corresponding share is higher: BMI was observed for 3,264 individuals (40%) at the last measurement point. If attrition is systematically related to body weight or other relevant characteristics, selection bias may arise. Multiple imputation by chained equations (MICE) was used to address missing data; however, this approach cannot fully eliminate potential bias resulting from non-random attrition. This is particularly relevant for the last two measurement points, at which BMI was observed for only 55% and 40% of the analytic sample, respectively. Descriptive results and the within-person variation used by the fixed-effects models therefore rest to a larger extent on imputed values in the second half of the observation period; descriptive results based on observed cases are provided for comparison (Table A7, Figures A1 and A2). We did not apply NEPS’s nonresponse-adjusted longitudinal weights [45]. These weights are calibrated on a restricted set of sociodemographic variables with known population benchmarks and cannot correct for selective attrition on variables such as personality traits or health status, for which no population distribution is available. Moreover, combining these weights with our multiply imputed data would require a weighted imputation procedure that was not implemented here. Consistent with the exploratory aim of this study, the results describe associations within the analytic sample rather than population-representative estimates for Germany, and representativeness may decrease over time as a result of selective attrition that these weights would only partially address. Moreover, fixed-effects models are robust to selection on time-constant individual characteristics, but remain sensitive to attrition related to the trajectory of BMI itself, which nonresponse weights could not address either.

Second, not all relevant variables were measured in every wave. In particular, alcohol consumption and sports participation at the first two measurement points were imputed from the third; a sensitivity analysis restricted to measurement points 3 to 6 yielded largely consistent results for the within-person associations (the employment coefficient was no longer significant, and the linear time trend was smaller because the largest increase occurs between the first two measurement points). In addition, time was modelled linearly in the main models; a specification with measurement-point dummies (Table A8) supports this assumption for the within-person associations and the education gradient, but shows that the overall increase in BMI was concentrated in the first years and the sex difference at the last measurement point. Third, physical and mental health and risk-taking were assessed after the baseline; their associations with BMI therefore cannot be interpreted as determinants, and the physical health score in particular may be a consequence of body weight. Fourth, individuals without any information on body weight and those with a BMI below 18.5 kg/m² were excluded (even if a quite limited number of cases) and possible selection on unobserved characteristics cannot be ruled out. Fixed-effects models remove bias from stable individual characteristics, but not from sample restrictions based on the outcome itself.

Finally, body weight and height were self-reported rather than objectively measured. Self-reported height and weight differ systematically from measured values: weight tends to be under-reported and height over-reported, so self-reported BMI is on average somewhat lower than measured BMI, particularly among individuals with higher body weight [46]. This should be considered when comparing our estimates with those based on measured anthropometric data, though it does not necessarily bias associations between BMI and other variables if the degree of misreporting is unrelated to those variables.

### Implications

The findings of this study have several implications for public health practice, future research, and policy. First, they help identify population groups that may be at elevated risk of overweight, obesity, and long-term weight gain. In particular, individuals with lower educational attainment exhibit higher BMI values and, according to the main model, somewhat more pronounced weight increases over time. These findings suggest that preventive interventions may benefit from targeting socioeconomic inequalities in health and health-related behaviors, in line with broader recommendations on addressing the social determinants of health [47] and with policy approaches specifically proposed to prevent and manage obesity, such as improving access to obesity management within primary care, restricting the marketing of unhealthy food, and strengthening health-promoting environments [48].

Second, the results highlight the potential relevance of psychological characteristics for understanding body weight and weight-related behaviors. Personality traits such as conscientiousness, extraversion, and neuroticism are associated with BMI, even after accounting for a broad range of sociodemographic and behavioral factors. These findings suggest that psychological factors may be useful to consider when designing and implementing prevention and treatment programs, particularly with regard to adherence and long-term behavior change.

Third, physical activity emerges as one of the most consistent factors associated with lower BMI, both between individuals and over time within individuals. This finding reinforces existing evidence on the importance of promoting regular physical activity as a central component of obesity prevention strategies. Public health initiatives that facilitate participation in sports and other forms of physical activity may therefore be worthwhile, although our observational data cannot establish that more physical activity causes lower body weight.

Finally, the negative cross-sectional association between alcohol consumption and BMI should not be interpreted as a protective effect of alcohol. Evidence on alcohol intake and body weight is inconsistent, and cross-sectional studies in particular often report null or inverse associations [11]. Such patterns are likely to reflect unobserved confounding, differences in physical activity and diet, and selective abstention among individuals with poorer health or higher body weight (“sick quitters”). The alcohol association is small but positive in the fixed-effects models for BMI (Table 3; not significant for overweight/obesity), meaning that higher consumption is associated with somewhat stronger weight increases over time.

## Conclusion

This study provides an exploratory overview of how socioeconomic, behavioral, and psychological factors are jointly associated with body weight and weight change among adults in Germany. While the broad mechanisms linking these factors to obesity are well documented [6], less is known about their relative and joint importance within a single, long-running German cohort. Future work should build on this by testing targeted hypotheses about specific factors and pathways, and by evaluating how existing knowledge on the determinants of obesity can be translated into effective prevention and intervention strategies for this population.

## Supplementary Information

Supplementary Material 1.

Supplementary Material 2.

## Data Availability

The dataset supporting the conclusions of this article is available in the NEPS data repository (http://dx.doi.org/10.5157/NEPS:SC6:17.0.0) after registration as a researcher. Complete Stata do-files are available from the author upon request.

## Footnotes

1 This paper uses data from the National Educational Panel Study. The NEPS is carried out by the Leibniz Institute for Educational Trajectories (LIfBi, Germany) in cooperation with a nationwide network.

## Abbreviations

BfDI: Federal Commissioner for Data Protection and Freedom of Information
BFI-10: Big Five Inventory, 10-item short version
BMI: Body mass index
CAPI: Computer-assisted personal interviewing
CATI: Computer-assisted telephone interviewing
FE: Fixed effects
GDPR: General Data Protection Regulation
HEE: Higher education entrance qualification
HH: Household
IRB: Institutional Review Board
ISEI: International Socio-Economic Index of Occupational Status
KMK: Standing Conference of the Ministers of Education and Cultural Affairs of the Länder
LIfBi: Leibniz Institute for Educational Trajectories
MICE: Multiple imputation by chained equations
NEPS: National Educational Panel Study
OLS: Ordinary least squares
SC6: Starting Cohort 6 (adult cohort)
SD: Standard deviation
SF-12: 12-item Short Form Health Survey
SOEP: German Socio-Economic Panel
WHO: World Health Organization
