# Supplementary material for: Socioeconomic, behavioral, and psychological correlates of body weight and weight gain: evidence from a 12-year German panel study
Source: BMC Public Health. 2026 Oct 31;26:3146. doi: 10.1186/s12889-026-30138-w (PMC13723097; doi:10.1186/s12889-026-30138-w)
Supplement: Supplementary file 1 [file 12889_2026_30138_MOESM2_ESM.docx]

Online Appendix

Table A1: Descriptives for time-variant variables by survey time point

|  | mean | sd | median | min | max |
| --- | --- | --- | --- | --- | --- |
| BMI (T1) | 26.0 | 4.39 | 25.3 | 18.5 | 45.0 |
| BMI (T2) | 26.4 | 4.64 | 25.7 | 18.5 | 45.0 |
| BMI (T3) | 26.7 | 4.65 | 26.0 | 18.5 | 45.0 |
| BMI (T4) | 26.7 | 4.68 | 26.0 | 18.5 | 45.0 |
| BMI (T5) | 26.8 | 4.69 | 26.0 | 18.5 | 45.0 |
| BMI (T6) | 27.1 | 4.73 | 26.4 | 18.5 | 45.0 |
| Log. HH Income (T1) | 8.03 | 0.54 | 8.07 | 5.99 | 9.80 |
| Log. HH Income (T2) | 8.11 | 0.53 | 8.16 | 5.99 | 9.80 |
| Log. HH Income (T3) | 8.16 | 0.54 | 8.22 | 5.99 | 9.80 |
| Log. HH Income (T4) | 8.21 | 0.55 | 8.29 | 5.99 | 9.80 |
| Log. HH Income (T5) | 8.23 | 0.56 | 8.29 | 5.99 | 9.80 |
| Log. HH Income (T6) | 8.29 | 0.56 | 8.34 | 6.21 | 9.80 |
| Employed (T1) | 0.90 | 0.31 | 1 | 0 | 1 |
| Employed (T2) | 0.90 | 0.30 | 1 | 0 | 1 |
| Employed (T3) | 0.90 | 0.30 | 1 | 0 | 1 |
| Employed (T4) | 0.88 | 0.32 | 1 | 0 | 1 |
| Employed (T5) | 0.85 | 0.36 | 1 | 0 | 1 |
| Employed (T6) | 0.78 | 0.42 | 1 | 0 | 1 |
| Unemployment reported (T1) | 0.091 | 0.29 | 0 | 0 | 1 |
| Unemployment reported (T2) | 0.083 | 0.28 | 0 | 0 | 1 |
| Unemployment reported (T3) | 0.079 | 0.27 | 0 | 0 | 1 |
| Unemployment reported (T4) | 0.080 | 0.27 | 0 | 0 | 1 |
| Unemployment reported (T5) | 0.088 | 0.28 | 0 | 0 | 1 |
| Unemployment reported (T6) | 0.070 | 0.25 | 0 | 0 | 1 |
| ISEI (T1) | 52.9 | 20.7 | 54.5 | 11.0 | 89.0 |
| ISEI (T2) | 51.1 | 21.3 | 52.7 | 11.0 | 89.0 |
| ISEI (T3) | 51.3 | 21.1 | 52.7 | 11.0 | 89.0 |
| ISEI (T4) | 51.4 | 21.0 | 52.7 | 11.0 | 89.0 |
| ISEI (T5) | 51.3 | 21.0 | 52.7 | 11.0 | 89.0 |
| ISEI (T6) | 51.4 | 20.8 | 52.5 | 11.0 | 89.0 |
| Living together with partner (T1) | 0.87 | 0.33 | 1 | 0 | 1 |
| Living together with partner (T2) | 0.88 | 0.33 | 1 | 0 | 1 |
| Living together with partner (T3) | 0.87 | 0.34 | 1 | 0 | 1 |
| Living together with partner (T4) | 0.86 | 0.34 | 1 | 0 | 1 |
| Living together with partner (T5) | 0.86 | 0.34 | 1 | 0 | 1 |
| Living together with partner (T6) | 0.86 | 0.35 | 1 | 0 | 1 |
| Living together with children (T1) | 0.63 | 0.48 | 1 | 0 | 1 |
| Living together with children (T2) | 0.60 | 0.49 | 1 | 0 | 1 |
| Living together with children (T3) | 0.54 | 0.50 | 1 | 0 | 1 |
| Living together with children (T4) | 0.50 | 0.50 | 1 | 0 | 1 |
| Living together with children (T5) | 0.49 | 0.50 | 0 | 0 | 1 |
| Living together with children (T6) | 0.40 | 0.49 | 0 | 0 | 1 |
| Number of people in the HH (T1) | 2.95 | 1.27 | 3 | 1 | 8 |
| Number of people in the HH (T2) | 2.84 | 1.23 | 3 | 1 | 8 |
| Number of people in the HH (T3) | 2.71 | 1.23 | 2 | 1 | 8 |
| Number of people in the HH (T4) | 2.62 | 1.21 | 2 | 1 | 8 |
| Number of people in the HH (T5) | 2.59 | 1.20 | 2 | 1 | 8 |
| Number of people in the HH (T6) | 2.42 | 1.12 | 2 | 1 | 8 |
| Living in East Germany (T1) | 0.20 | 0.40 | 0 | 0 | 1 |
| Living in East Germany (T2) | 0.20 | 0.40 | 0 | 0 | 1 |
| Living in East Germany (T3) | 0.20 | 0.40 | 0 | 0 | 1 |
| Living in East Germany (T4) | 0.20 | 0.40 | 0 | 0 | 1 |
| Living in East Germany (T5) | 0.20 | 0.40 | 0 | 0 | 1 |
| Living in East Germany (T6) | 0.20 | 0.40 | 0 | 0 | 1 |
| Life satisfaction (T1) | 7.55 | 1.53 | 8 | 0 | 10 |
| Life satisfaction (T2) | 7.56 | 1.52 | 8 | 0 | 10 |
| Life satisfaction (T3) | 7.64 | 1.39 | 8 | 0 | 10 |
| Life satisfaction (T4) | 7.60 | 1.37 | 8 | 0 | 10 |
| Life satisfaction (T5) | 7.36 | 1.45 | 8 | 0 | 10 |
| Life satisfaction (T6) | 7.49 | 1.43 | 8 | 0 | 10 |
| Smoking (T1) | 0.30 | 0.46 | 0 | 0 | 1 |
| Smoking (T2) | 0.29 | 0.45 | 0 | 0 | 1 |
| Smoking (T3) | 0.27 | 0.44 | 0 | 0 | 1 |
| Smoking (T4) | 0.26 | 0.44 | 0 | 0 | 1 |
| Smoking (T5) | 0.25 | 0.43 | 0 | 0 | 1 |
| Smoking (T6) | 0.25 | 0.43 | 0 | 0 | 1 |
| Frequency alcohol consumption (T1) | 3.16 | 1.38 | 3 | 1 | 6 |
| Frequency alcohol consumption (T2) | 3.16 | 1.38 | 3 | 1 | 6 |
| Frequency alcohol consumption (T3) | 3.16 | 1.38 | 3 | 1 | 6 |
| Frequency alcohol consumption (T4) | 3.17 | 1.42 | 3 | 1 | 6 |
| Frequency alcohol consumption (T5) | 3.20 | 1.44 | 3 | 1 | 6 |
| Frequency alcohol consumption (T6) | 3.02 | 1.43 | 3 | 1 | 6 |
| Frequency doing sports (T1) | 2.99 | 1.19 | 3 | 1 | 5 |
| Frequency doing sports (T2) | 2.99 | 1.19 | 3 | 1 | 5 |
| Frequency doing sports (T3) | 2.99 | 1.19 | 3 | 1 | 5 |
| Frequency doing sports (T4) | 3.00 | 1.19 | 3 | 1 | 5 |
| Frequency doing sports (T5) | 3.02 | 1.22 | 3 | 1 | 5 |
| Frequency doing sports (T6) | 2.98 | 1.25 | 3 | 1 | 5 |
| Observations | 8209 | | | | |

Source: NEPS SC6, imputed data (N=8,209; M=50). Mean ISEI is lower in the imputed than in the observed data from T2 onwards (e.g., 51.1 vs. 53.1 at T2; compare Table A7). ISEI is observed only for respondents who took part in a wave and reported an employment spell; the imputed values therefore concern predominantly respondents who were not employed or did not participate, who plausibly have lower occupational status on average.

Table A2: Baseline regression when omitting risk, mental and physical health

|  | M5 | M6 |
| --- | --- | --- |
|  | BMI (continuous) | Being overweight/obese |
| Log. HH income° | -0.226^**^ | -0.013 |
|  | [-0.368,-0.084] | [-0.030,0.004] |
| Employed | -0.451^*^ | -0.027 |
|  | [-0.843,-0.060] | [-0.068,0.014] |
| Unemployment episode reported | 0.374 | 0.016 |
|  | [-0.049,0.797] | [-0.027,0.058] |
| ISEI° | -0.214^**^ | -0.026^***^ |
|  | [-0.344,-0.084] | [-0.041,-0.011] |
| Living together with partner | 0.346 | 0.038 |
|  | [-0.028,0.721] | [-0.003,0.079] |
| Living together with children | -0.189 | -0.011 |
|  | [-0.483,0.105] | [-0.044,0.023] |
| Number of people in the HH | 0.138^*^ | 0.016^*^ |
|  | [0.019,0.257] | [0.002,0.030] |
| Living in East Germany | 0.201 | 0.039^**^ |
|  | [-0.037,0.440] | [0.011,0.068] |
| Life satisfaction° | -0.139^*^ | -0.007 |
|  | [-0.252,-0.025] | [-0.020,0.005] |
| Smoking | -0.865^***^ | -0.069^***^ |
|  | [-1.085,-0.645] | [-0.095,-0.043] |
| Frequency alcohol consumption° | -0.470^***^ | -0.035^***^ |
|  | [-0.580,-0.360] | [-0.049,-0.022] |
| Frequency doing sports° | -0.456^***^ | -0.043^***^ |
|  | [-0.567,-0.344] | [-0.056,-0.031] |
| Female respondent | -1.353^***^ | -0.178^***^ |
|  | [-1.562,-1.144] | [-0.202,-0.153] |
| Age at baseline | 0.073^***^ | 0.009^***^ |
|  | [0.060,0.087] | [0.007,0.010] |
| Migration background | 0.137 | 0.020 |
|  | [-0.113,0.387] | [-0.009,0.049] |
| HEE (Abitur) | -0.454^***^ | -0.045^**^ |
|  | [-0.722,-0.185] | [-0.078,-0.013] |
| Tertiary | -0.618^***^ | -0.064^***^ |
|  | [-0.901,-0.335] | [-0.097,-0.031] |
| Social origin° | -0.300^***^ | -0.032^***^ |
|  | [-0.409,-0.191] | [-0.046,-0.019] |
| B5: Extraversion° | 0.253^***^ | 0.025^***^ |
|  | [0.142,0.364] | [0.013,0.038] |
| B5: Agreeableness° | -0.114^*^ | -0.015^*^ |
|  | [-0.216,-0.012] | [-0.027,-0.003] |
| B5: Conscientiousness° | -0.694^***^ | -0.057^***^ |
|  | [-0.805,-0.584] | [-0.070,-0.045] |
| B5: Neuroticism° | -0.217^***^ | -0.018^**^ |
|  | [-0.330,-0.104] | [-0.031,-0.004] |
| B5: Openness° | 0.065 | -0.001 |
|  | [-0.039,0.168] | [-0.013,0.011] |
| Self-worth° | -0.066 | -0.001 |
|  | [-0.199,0.067] | [-0.015,0.014] |
| Constant | 23.663^***^ | 0.229^***^ |
|  | [22.814,24.512] | [0.128,0.330] |
| R² (%) | 12.89 | 10.56 |
| Observations | 8209 | 8209 |

Source: NEPS SC6, imputed data (N=8,209; M=50). 95% confidence intervals included, based on robust standard errors. Omitting risk, mental, and physical health.	° Variable is z-standardized.	^*^ *p* < 0.05, ^**^ *p* < 0.01, ^***^ *p* < 0.001

Table A3: FE regressions when excluding T1 and T2

|  | M7 | M8 |
| --- | --- | --- |
|  | BMI (continuous) | Being overweight/obese |
| Years since baseline | 0.033 | 0.004 |
|  | [-0.019,0.085] | [-0.002,0.010] |
| Log. HH income° | 0.085^*^ | 0.008 |
|  | [0.009,0.160] | [-0.004,0.020] |
| Employed | -0.118 | -0.003 |
|  | [-0.245,0.008] | [-0.023,0.017] |
| Unemployment episode reported | 0.151 | 0.011 |
|  | [-0.000,0.302] | [-0.013,0.034] |
| ISEI° | -0.073 | -0.008 |
|  | [-0.200,0.054] | [-0.026,0.011] |
| Living together with partner | 0.351^*^ | 0.052^*^ |
|  | [0.066,0.636] | [0.007,0.096] |
| Living together with children | 0.009 | 0.011 |
|  | [-0.132,0.150] | [-0.016,0.037] |
| Number of people in the HH | -0.020 | -0.002 |
|  | [-0.089,0.048] | [-0.014,0.011] |
| Living in East Germany | 0.230 | -0.029 |
|  | [-0.125,0.586] | [-0.093,0.034] |
| Life satisfaction° | 0.051^*^ | 0.004 |
|  | [0.012,0.090] | [-0.002,0.010] |
| Smoking | -0.356^***^ | -0.034^**^ |
|  | [-0.500,-0.213] | [-0.057,-0.010] |
| Frequency alcohol consumption° | 0.075^*^ | 0.005 |
|  | [0.010,0.140] | [-0.005,0.014] |
| Frequency doing sports° | -0.142^***^ | -0.012^***^ |
|  | [-0.187,-0.097] | [-0.018,-0.005] |
| Female respondent # Years since baseline | 0.089^*^ | 0.007 |
|  | [0.012,0.166] | [-0.001,0.015] |
| Years since baseline # Age at baseline (centred at 44) | -0.005^***^ | -0.001^***^ |
|  | [-0.007,-0.003] | [-0.001,-0.000] |
| Migration background # Years since baseline | 0.009 | 0.000 |
|  | [-0.028,0.046] | [-0.005,0.005] |
| HEE (Abitur) # Years since baseline | -0.007 | -0.001 |
|  | [-0.044,0.029] | [-0.007,0.004] |
| Tertiary # Years since baseline | -0.019 | -0.000 |
|  | [-0.052,0.014] | [-0.006,0.005] |
| Years since baseline # Social origin° | -0.006 | 0.000 |
|  | [-0.020,0.009] | [-0.002,0.002] |
| Years since baseline # B5: Extraversion° | 0.014 | 0.002 |
|  | [-0.000,0.029] | [-0.000,0.004] |
| Years since baseline # B5: Agreeableness° | -0.002 | 0.000 |
|  | [-0.017,0.014] | [-0.002,0.002] |
| Years since baseline # B5: Conscientiousness° | -0.000 | 0.001 |
|  | [-0.014,0.013] | [-0.001,0.003] |
| Years since baseline # B5: Neuroticism° | 0.004 | 0.001 |
|  | [-0.010,0.019] | [-0.001,0.003] |
| Years since baseline # B5: Openness° | -0.005 | -0.001 |
|  | [-0.018,0.008] | [-0.003,0.001] |
| Years since baseline # Self-worth° | 0.003 | 0.001 |
|  | [-0.014,0.021] | [-0.002,0.003] |
| Years since baseline # Risk taking° | -0.001 | -0.000 |
|  | [-0.014,0.012] | [-0.002,0.002] |
| Years since baseline # Mental health score° | -0.016^*^ | -0.002 |
|  | [-0.032,-0.000] | [-0.004,0.001] |
| Years since baseline # Physical health score° | 0.013 | 0.002 |
|  | [-0.003,0.028] | [-0.001,0.004] |
| Constant | 26.072^***^ | 0.514^***^ |
|  | [25.680,26.463] | [0.453,0.575] |
| Observations | 32836 | 32836 |

Source: NEPS SC6, imputed data (N=8,209; M=50). 95% confidence intervals included, based on robust standard errors. Measurement points 1 and 2 are excluded. Age at baseline is centred at 44 years; the time coefficient refers to a 44-year-old man with low education, no migration background, and average values on all standardized variables.	° Variable is z-standardized.	^*^ *p* < 0.05, ^**^ *p* < 0.01, ^***^ *p* < 0.001

Table A4: FE regressions by sex

|  | M9 | M10 | M11 | M12 |
| --- | --- | --- | --- | --- |
|  | BMI (male) | BMI (female) | Being overweight/obese (male) | Being overweight/obese (female) |
| Years since baseline | 0.072^***^ | 0.106^***^ | 0.006^***^ | 0.009^***^ |
|  | [0.049,0.096] | [0.078,0.135] | [0.003,0.009] | [0.006,0.012] |
| Log. HH income° | 0.049 | 0.130^**^ | 0.000 | 0.014^*^ |
|  | [-0.029,0.126] | [0.042,0.218] | [-0.013,0.013] | [0.000,0.028] |
| Employed | -0.129 | -0.125 | -0.001 | -0.010 |
|  | [-0.295,0.037] | [-0.272,0.021] | [-0.025,0.023] | [-0.033,0.012] |
| Unemployment episode reported | -0.017 | 0.135 | 0.002 | 0.012 |
|  | [-0.201,0.167] | [-0.033,0.303] | [-0.024,0.028] | [-0.012,0.036] |
| ISEI° | -0.028 | -0.075 | -0.007 | -0.009 |
|  | [-0.142,0.085] | [-0.225,0.076] | [-0.023,0.009] | [-0.029,0.010] |
| Living together with partner | 0.159 | 0.358^*^ | 0.017 | 0.040^*^ |
|  | [-0.123,0.442] | [0.068,0.648] | [-0.022,0.056] | [0.002,0.078] |
| Living together with children | 0.030 | -0.068 | 0.006 | -0.002 |
|  | [-0.123,0.183] | [-0.227,0.091] | [-0.020,0.031] | [-0.027,0.023] |
| Number of people in the HH | -0.008 | -0.028 | 0.003 | -0.004 |
|  | [-0.074,0.058] | [-0.114,0.058] | [-0.008,0.014] | [-0.017,0.008] |
| Living in East Germany | -0.119 | -0.193 | -0.043 | -0.046 |
|  | [-0.525,0.288] | [-0.752,0.367] | [-0.124,0.038] | [-0.130,0.037] |
| Life satisfaction° | 0.049^*^ | 0.027 | 0.006 | -0.001 |
|  | [0.001,0.098] | [-0.018,0.071] | [-0.002,0.014] | [-0.007,0.006] |
| Smoking | -0.509^***^ | -0.524^***^ | -0.036^**^ | -0.058^***^ |
|  | [-0.672,-0.346] | [-0.707,-0.341] | [-0.062,-0.010] | [-0.087,-0.029] |
| Frequency alcohol consumption° | 0.142^**^ | 0.026 | 0.014^*^ | 0.000 |
|  | [0.055,0.228] | [-0.088,0.140] | [0.001,0.027] | [-0.015,0.016] |
| Frequency doing sports° | -0.103^**^ | -0.152^***^ | -0.009 | -0.011^*^ |
|  | [-0.167,-0.040] | [-0.222,-0.083] | [-0.018,0.001] | [-0.021,-0.002] |
| Years since baseline # Age at baseline (centred at 44) | -0.004^***^ | -0.004^***^ | -0.000^***^ | -0.000^**^ |
|  | [-0.006,-0.003] | [-0.005,-0.002] | [-0.001,-0.000] | [-0.001,-0.000] |
| Migration background # Years since baseline | 0.016 | -0.003 | 0.001 | -0.001 |
|  | [-0.012,0.043] | [-0.030,0.024] | [-0.003,0.005] | [-0.005,0.003] |
| HEE (Abitur) # Years since baseline | -0.002 | -0.008 | 0.001 | -0.000 |
|  | [-0.027,0.022] | [-0.035,0.019] | [-0.003,0.005] | [-0.004,0.003] |
| Tertiary # Years since baseline | -0.019 | -0.027^*^ | 0.001 | -0.002 |
|  | [-0.040,0.003] | [-0.053,-0.001] | [-0.002,0.004] | [-0.006,0.001] |
| Years since baseline # Social origin° | 0.001 | -0.006 | 0.001 | -0.001 |
|  | [-0.008,0.011] | [-0.017,0.006] | [-0.001,0.002] | [-0.002,0.001] |
| Years since baseline # B5: Extraversion° | 0.005 | 0.003 | 0.001 | 0.001 |
|  | [-0.006,0.015] | [-0.010,0.017] | [-0.001,0.002] | [-0.001,0.003] |
| Years since baseline # B5: Agreeableness° | -0.003 | 0.007 | 0.000 | 0.001 |
|  | [-0.013,0.006] | [-0.005,0.018] | [-0.001,0.002] | [-0.000,0.003] |
| Years since baseline # B5: Conscientiousness° | -0.006 | -0.006 | 0.000 | 0.000 |
|  | [-0.015,0.003] | [-0.017,0.005] | [-0.001,0.002] | [-0.002,0.002] |
| Years since baseline # B5: Neuroticism° | 0.005 | -0.004 | 0.001 | 0.000 |
|  | [-0.005,0.014] | [-0.016,0.008] | [-0.001,0.002] | [-0.002,0.002] |
| Years since baseline # B5: Openness° | -0.002 | 0.007 | 0.000 | -0.000 |
|  | [-0.011,0.006] | [-0.005,0.018] | [-0.001,0.002] | [-0.002,0.001] |
| Years since baseline # Self-worth° | 0.012 | -0.010 | 0.001 | 0.000 |
|  | [-0.000,0.024] | [-0.021,0.002] | [-0.001,0.003] | [-0.001,0.002] |
| Years since baseline # Risk taking° | 0.002 | -0.002 | 0.001 | 0.000 |
|  | [-0.008,0.012] | [-0.014,0.010] | [-0.001,0.002] | [-0.001,0.002] |
| Years since baseline # Mental health score° | -0.014^*^ | -0.011 | -0.002^*^ | -0.002 |
|  | [-0.024,-0.003] | [-0.024,0.002] | [-0.003,-0.000] | [-0.003,0.000] |
| Years since baseline # Physical health score° | -0.002 | -0.007 | 0.001 | 0.001 |
|  | [-0.013,0.010] | [-0.020,0.006] | [-0.000,0.003] | [-0.001,0.002] |
| Constant | 26.928^***^ | 25.497^***^ | 0.627^***^ | 0.461^***^ |
|  | [26.577,27.278] | [25.130,25.864] | [0.573,0.682] | [0.408,0.514] |
| Observations | 23934 | 25320 | 23934 | 25320 |

Source: NEPS SC6, imputed data (N=8,209; M=50). 95% confidence intervals included, based on robust standard errors. Age at baseline is centred at 44 years; the time coefficients refer to a 44-year-old with low education, no migration background, and average values on all standardized variables.	° Variable is z-standardized.	^*^ *p* < 0.05, ^**^ *p* < 0.01, ^***^ *p* < 0.001

Table A5: Baseline regressions by sex

|  | M13 | M14 | M15 | M16 |
| --- | --- | --- | --- | --- |
|  | BMI (male) | BMI (female) | Being overweight/obese (male) | Being overweight/obese (female) |
| Log. HH income° | 0.057 | -0.368^***^ | 0.020 | -0.036^**^ |
|  | [-0.126,0.240] | [-0.570,-0.166] | [-0.004,0.044] | [-0.058,-0.014] |
| Employed | -0.394 | 0.082 | -0.049 | 0.020 |
|  | [-1.090,0.302] | [-0.379,0.542] | [-0.124,0.026] | [-0.029,0.070] |
| Unemployment episode reported | 0.242 | 0.352 | -0.007 | 0.018 |
|  | [-0.404,0.888] | [-0.181,0.885] | [-0.074,0.061] | [-0.036,0.072] |
| ISEI° | 0.033 | -0.386^***^ | -0.014 | -0.033^**^ |
|  | [-0.127,0.193] | [-0.589,-0.182] | [-0.035,0.008] | [-0.054,-0.011] |
| Living together with partner | 0.465 | 0.453 | 0.039 | 0.059^*^ |
|  | [-0.079,1.009] | [-0.090,0.996] | [-0.026,0.104] | [0.005,0.114] |
| Living together with children | -0.179 | -0.150 | 0.001 | -0.018 |
|  | [-0.549,0.191] | [-0.592,0.291] | [-0.047,0.049] | [-0.065,0.030] |
| Number of people in the HH | 0.164^*^ | 0.063 | 0.017 | 0.009 |
|  | [0.017,0.311] | [-0.116,0.242] | [-0.003,0.037] | [-0.010,0.029] |
| Living in East Germany | 0.074 | 0.376^*^ | 0.027 | 0.053^**^ |
|  | [-0.229,0.378] | [0.027,0.726] | [-0.013,0.067] | [0.014,0.093] |
| Life satisfaction° | 0.081 | -0.108 | 0.009 | -0.007 |
|  | [-0.085,0.247] | [-0.267,0.052] | [-0.011,0.028] | [-0.024,0.010] |
| Smoking | -0.792^***^ | -1.004^***^ | -0.067^***^ | -0.075^***^ |
|  | [-1.067,-0.518] | [-1.331,-0.676] | [-0.101,-0.032] | [-0.112,-0.037] |
| Frequency alcohol consumption° | -0.303^***^ | -0.478^***^ | -0.019^*^ | -0.040^***^ |
|  | [-0.444,-0.162] | [-0.642,-0.313] | [-0.037,-0.001] | [-0.059,-0.020] |
| Frequency doing sports° | -0.457^***^ | -0.313^***^ | -0.044^***^ | -0.032^***^ |
|  | [-0.600,-0.313] | [-0.495,-0.131] | [-0.062,-0.025] | [-0.051,-0.013] |
| Age at baseline | 0.031^***^ | 0.036^***^ | 0.005^***^ | 0.005^***^ |
|  | [0.013,0.050] | [0.015,0.056] | [0.003,0.008] | [0.003,0.008] |
| Migration background | 0.088 | 0.137 | 0.029 | 0.007 |
|  | [-0.235,0.412] | [-0.214,0.488] | [-0.014,0.072] | [-0.031,0.046] |
| Low education | Ref. | Ref. | Ref. | Ref. |
| HEE (Abitur) | -0.260 | -0.245 | -0.030 | -0.029 |
|  | [-0.617,0.097] | [-0.614,0.125] | [-0.078,0.017] | [-0.072,0.014] |
| Tertiary | -0.538^**^ | -0.399 | -0.051^*^ | -0.058^*^ |
|  | [-0.891,-0.185] | [-0.815,0.017] | [-0.096,-0.005] | [-0.105,-0.011] |
| Social origin° | -0.271^***^ | -0.218^**^ | -0.031^**^ | -0.024^**^ |
|  | [-0.405,-0.137] | [-0.380,-0.056] | [-0.050,-0.013] | [-0.042,-0.007] |
| B5: Extraversion° | 0.323^***^ | 0.151 | 0.035^***^ | 0.014 |
|  | [0.175,0.471] | [-0.009,0.311] | [0.017,0.054] | [-0.004,0.031] |
| B5: Agreeableness° | -0.064 | -0.136 | -0.009 | -0.018^*^ |
|  | [-0.195,0.068] | [-0.280,0.009] | [-0.026,0.007] | [-0.035,-0.002] |
| B5: Conscientiousness° | -0.472^***^ | -0.812^***^ | -0.041^***^ | -0.066^***^ |
|  | [-0.607,-0.338] | [-0.979,-0.646] | [-0.058,-0.024] | [-0.084,-0.049] |
| B5: Neuroticism° | -0.198^*^ | -0.340^***^ | -0.013 | -0.027^**^ |
|  | [-0.353,-0.044] | [-0.506,-0.174] | [-0.032,0.006] | [-0.046,-0.009] |
| B5: Openness° | -0.064 | 0.181^*^ | -0.018^*^ | 0.016 |
|  | [-0.198,0.071] | [0.035,0.327] | [-0.035,-0.001] | [-0.001,0.032] |
| Self-worth° | 0.040 | -0.042 | 0.006 | -0.001 |
|  | [-0.138,0.219] | [-0.230,0.146] | [-0.017,0.028] | [-0.021,0.019] |
| Risk taking° | -0.108 | -0.233^**^ | -0.011 | -0.015 |
|  | [-0.259,0.042] | [-0.399,-0.068] | [-0.030,0.008] | [-0.033,0.004] |
| Mental health score° | -0.024 | 0.033 | 0.008 | 0.007 |
|  | [-0.197,0.149] | [-0.139,0.204] | [-0.012,0.029] | [-0.012,0.026] |
| Physical health score° | -0.994^***^ | -1.190^***^ | -0.074^***^ | -0.095^***^ |
|  | [-1.154,-0.834] | [-1.358,-1.022] | [-0.093,-0.056] | [-0.112,-0.078] |
| Constant | 25.285^***^ | 23.482^***^ | 0.377^***^ | 0.160^*^ |
|  | [24.059,26.511] | [22.257,24.706] | [0.225,0.529] | [0.022,0.299] |
| R² | 14.91 | 19.57 | 9.43 | 13.19 |
| Observations | 3989 | 4220 | 3989 | 4220 |

Source: NEPS SC6, imputed data (N=8,209; M=50). 95% confidence intervals included, based on robust standard errors. ° Variable is z-standardized.	^*^ *p* < 0.05, ^**^ *p* < 0.01, ^***^ *p* < 0.001

Table A6: Variable and instrument overview in the NEPS SC6

| **NEPS variable** | **Construct** | **Wave(s)** | **Original question text (English)** | **Response options (Original, before recoding)** |
| --- | --- | --- | --- | --- |
| t520000 | Body weight (self-reported) | every wave | “And how much do you weigh?” | Open numeric (kg) |
| t520001 | Height (self-reported) | every wave | “What's your height?” | Open numeric (cm) |
| t700001 | Sex | background | “Which gender do you have?” | Male; Female |
| t70000y | Year of birth | background | “Please specify your date of birth!” | Day / Month / Year (open) |
| t400500_g1v1 | Migration background (generation status) | derived, background | Generation status (derived from country-of-birth and parental country-of-birth items) | 0 no migrant background; 1 1st generation; 2 1.5th generation; 3 2nd generation; 4 2.25th generation; 5 2.5th generation; 6 2.75th generation; 7 3rd generation; 8 3.25th generation; 9 3.5th generation. Recoded: 1–6 = migration background (respondent or at least one parent born abroad); 0 and 7–9 = no migration background |
| t525008 | Smoking status | every wave | “Do you smoke at the moment – even if only occasionally?” | 1 yes, daily; 2 yes, occasionally; 3 no, not anymore; 4 have never smoked |
| t525209 | Alcohol consumption frequency | every wave | Alcohol consumption frequency, last 12 months | 1 never; 2 once a month or less; 3 twice or three times a month; 4 once a week; 5 several times a week; 6 daily |
| t527102 | Sports participation frequency | every wave | “How often do you do sport?” | 1 never; 2 once a month or less; 3 several times a month or once a week; 4 several times a week; 5 almost daily or daily |
| t743021 | Living with partner | every wave | “And with whom do you currently live in a household? / with spouse / partner” | Yes; No |
| t743022 | Living with children | every wave | “And with whom do you currently live in a household? / with own children / foster children / adopted children” | Yes; No |
| t741001 | Household size | every wave | “Now some questions about your household. How many people currently live with you in your home, including yourself and any children?” | Open numeric |
| t751001_g1 | East/West Germany (place of residence) | every wave | Derived from: “Where do you live today? Please tell me the exact name of your town.” | West Germany; East Germany |
| t510010 | Household income | every wave | “Now let’s talk about all your household income: how much is your monthly household income today? Please enter the net amount, after deduction of taxes and social security contributions. Please include regular payments such as pensions, rent allowance, children’s and parental allowance, student financial aid (BAföG), educational maintenance allowance, unemployment benefit and so on!” | Open numeric (EUR) |
| t514001 | Life satisfaction | every wave | “First of all, I would like to ask you some questions about your current satisfaction with various aspects of your life. Please answer on a scale from 0 to 10. ‘0’ means that you are ‘completely unsatisfied’, ‘10’ means that you are ‘completely satisfied’. You can gradate your answer with the numbers in between. All in all, how satisfied are you with your life at the moment?” | 0 (completely dissatisfied) – 10 (completely satisfied) |
| ts23201_g14 | ISEI (highest ISEI of all job spells reported in the wave) | every wave, employment module | “Let’s start with the first job you had after [last interview]. Please tell me what occupation you had.” (occupation description, coded to ISEI) | Open text → coded occupational classification (ISEI-08); carried forward within continuing spells |
| ts2312c | Employment status (employed at interview date) | every wave, employment module | Derived from the employment spell data: indicator of whether the reported employment spell is still ongoing at the interview date (“Andauern der Erwerbsepisode”). Respondents interviewed without an ongoing employment spell were coded 0. Not a single directly asked question. | Spell ongoing / spell ended (coded) → binary |
| t66003a_g1 | Self-worth | wave 3 | For details refer to the main instrument documentation: https://doi.org/10.1024//0170-1789.24.1.3 | Does not apply at all – applies completely (5-point scale) |
| t66800a_g1–t66800e_g1 | Big Five personality traits (Extraversion, Agreeableness, Conscientiousness, Neuroticism, Openness) | wave 4 | *Ten-item Big Five Inventory (BFI-10); item wording follows the published short-scale instrument and is not reproduced item-by-item here — see cited source in the maintext or https://doi.org/10.1016/j.jrp.2006.02.001* | 5-point agreement scale (does not apply at all – applies completely) |
| t731351_g2 | Father's education | first wave available (wave 3 or later) | “What is/was your father's (stepfather's / this person's) highest general school-leaving qualification?” | Categorical (school-leaving qualification levels) |
| t731301_g2 | Mother's education | first wave available (wave 3 or later) | “What is/was your mother's (stepmother's / this person's) highest general school-leaving qualification?” | Categorical (school-leaving qualification levels) |
| t731453_g14 | Father's occupation (→ ISEI) | first wave available (wave 3 or later) | “What did your father [stepfather / this person] work as back then?” | Open text → coded occupational classification (ISEI-08) |
| t731403_g14 | Mother's occupation (→ ISEI) | first wave available (wave 3 or later) | *Parallel to the previous item, but for the mother* | Open text → coded occupational classification (ISEI-08) |
| t731457 | Father's leadership position | first wave available (wave 3 or later) | “Did he [father] hold a management position?” | Yes; No |
| t515051 | Risk taking (general) | wave 8 | “How do you personally assess yourself: Are you generally willing to take risks or do you try to avoid risks?” | 0 (not willing to take risks at all) – 10 (very willing to take risks) |
| t515052 | Risk taking (financial investments) | wave 8 | “How would you rate your willingness to take risks in relation to financial investments?” | 0 – 10, same anchors |
| t515053 | Risk taking (leisure and sport) | wave 8 | “How would you rate your willingness to take risks in relation to leisure and sport?” | 0 – 10, same anchors |
| t515054 | Risk taking (professional career) | wave 8 | “How would you rate your willingness to take risks regarding your professional career?” | 0 – 10, same anchors |
| t521300_g1 | Physical health score | wave 11 | SF-12 Physical Component Score (SOEP norm-based score); derived from the SF-12 health survey items — see cited source | Standardized score (SOEP norm-based, M = 50, SD = 10) |
| t521300_g2 | Mental health score | wave 11 | SF-12 Mental Component Score (SOEP norm-based score); derived from the SF-12 health survey items — see cited source | Standardized score (SOEP norm-based, M = 50, SD = 10) |
| tx28101 | CASMIN classification | derived, education module | Derived classification of educational attainment (CASMIN scheme) | 0 no qualification; 1 Hauptschule w/o vocational qualification; 2 Hauptschule w/ vocational qualification; 3 Mittlere Reife w/o vocational qualification; 4 Mittlere Reife w/ vocational qualification; 5 higher education entrance qualification w/o vocational qualification; 6 higher education entrance qualification w/ vocational qualification; 7 university of applied sciences degree; 8 university degree |
| sptype = 27 | Unemployment in the 12 months before the interview | biography module | Derived from the retrospective life-course (biography) module, which records episodes (e.g., employment, unemployment, education) with start and end dates; episode type 27 denotes an unemployment episode. Coded 1 if an unemployment episode overlapped the 12 months before the interview date of the respective wave, otherwise 0. Not a single directly asked question. | Categorical episode type (coded) |

Note: Item wording follows the SUF-version survey instruments published by the Leibniz Institute for Educational Trajectories (LIfBi). The Big Five items (BFI-10) are not reproduced individually here; see the cited short-scale instrument source. Physical and mental health scores (SF-12) are derived scores; see the cited NEPS Survey Paper for the underlying item battery. https://www.neps-data.de/Data-Center/Data-and-Documentation/SC6-Adults/Documentation

Table A7: Descriptives for time-variant variables by survey time point, observed cases only (no imputation)

|  | mean | sd | median | min | max | N obs. |
| --- | --- | --- | --- | --- | --- | --- |
| BMI (T1) | 25.9 | 4.32 | 25.3 | 18.5 | 45.0 | 6,026 |
| BMI (T2) | 26.5 | 4.68 | 25.7 | 18.5 | 45.0 | 6,287 |
| BMI (T3) | 26.7 | 4.72 | 25.9 | 18.5 | 45.0 | 5,317 |
| BMI (T4) | 26.8 | 4.77 | 26.0 | 18.5 | 45.0 | 4,825 |
| BMI (T5) | 26.8 | 4.76 | 26.0 | 18.5 | 45.0 | 4,520 |
| BMI (T6) | 27.1 | 4.82 | 26.3 | 18.5 | 45.0 | 3,264 |
| Log. HH Income (T1) | 8.03 | 0.53 | 8.07 | 5.99 | 9.80 | 7,307 |
| Log. HH Income (T2) | 8.11 | 0.53 | 8.16 | 5.99 | 9.80 | 5,698 |
| Log. HH Income (T3) | 8.18 | 0.54 | 8.24 | 5.99 | 9.80 | 5,142 |
| Log. HH Income (T4) | 8.25 | 0.54 | 8.29 | 5.99 | 9.80 | 4,563 |
| Log. HH Income (T5) | 8.26 | 0.54 | 8.29 | 5.99 | 9.80 | 4,362 |
| Log. HH Income (T6) | 8.34 | 0.55 | 8.41 | 6.21 | 9.80 | 3,098 |
| Employed (T1) | 0.90 | 0.31 | 1 | 0 | 1 | 8,073 |
| Employed (T2) | 0.90 | 0.30 | 1 | 0 | 1 | 6,488 |
| Employed (T3) | 0.91 | 0.29 | 1 | 0 | 1 | 5,564 |
| Employed (T4) | 0.89 | 0.31 | 1 | 0 | 1 | 4,912 |
| Employed (T5) | 0.87 | 0.34 | 1 | 0 | 1 | 4,680 |
| Employed (T6) | 0.78 | 0.42 | 1 | 0 | 1 | 3,306 |
| Unemployment reported (T1) | 0.091 | 0.29 | 0 | 0 | 1 | 8,073 |
| Unemployment reported (T2) | 0.080 | 0.27 | 0 | 0 | 1 | 6,488 |
| Unemployment reported (T3) | 0.071 | 0.26 | 0 | 0 | 1 | 5,564 |
| Unemployment reported (T4) | 0.067 | 0.25 | 0 | 0 | 1 | 4,912 |
| Unemployment reported (T5) | 0.076 | 0.26 | 0 | 0 | 1 | 4,680 |
| Unemployment reported (T6) | 0.059 | 0.24 | 0 | 0 | 1 | 3,306 |
| ISEI (T1) | 53.4 | 20.6 | 54.5 | 11.6 | 89.0 | 7,522 |
| ISEI (T2) | 53.1 | 20.9 | 54.5 | 11.6 | 89.0 | 5,913 |
| ISEI (T3) | 53.9 | 20.5 | 55.2 | 11.7 | 89.0 | 5,085 |
| ISEI (T4) | 54.4 | 20.3 | 55.2 | 11.7 | 89.0 | 4,441 |
| ISEI (T5) | 54.8 | 20.1 | 56.0 | 11.7 | 89.0 | 4,192 |
| ISEI (T6) | 56.2 | 19.6 | 56.0 | 11.7 | 89.0 | 2,708 |
| Living together with partner (T1) | 0.90 | 0.30 | 1 | 0 | 1 | 6,988 |
| Living together with partner (T2) | 0.91 | 0.29 | 1 | 0 | 1 | 5,586 |
| Living together with partner (T3) | 0.92 | 0.28 | 1 | 0 | 1 | 4,721 |
| Living together with partner (T4) | 0.92 | 0.27 | 1 | 0 | 1 | 4,127 |
| Living together with partner (T5) | 0.92 | 0.27 | 1 | 0 | 1 | 3,921 |
| Living together with partner (T6) | 0.94 | 0.25 | 1 | 0 | 1 | 2,704 |
| Living together with children (T1) | 0.71 | 0.46 | 1 | 0 | 1 | 6,988 |
| Living together with children (T2) | 0.67 | 0.47 | 1 | 0 | 1 | 5,586 |
| Living together with children (T3) | 0.62 | 0.49 | 1 | 0 | 1 | 4,721 |
| Living together with children (T4) | 0.57 | 0.50 | 1 | 0 | 1 | 4,127 |
| Living together with children (T5) | 0.55 | 0.50 | 1 | 0 | 1 | 3,921 |
| Living together with children (T6) | 0.45 | 0.50 | 0 | 0 | 1 | 2,704 |
| Number of people in the HH (T1) | 2.95 | 1.27 | 3 | 1 | 8 | 8,068 |
| Number of people in the HH (T2) | 2.85 | 1.24 | 3 | 1 | 8 | 6,483 |
| Number of people in the HH (T3) | 2.72 | 1.22 | 3 | 1 | 8 | 5,563 |
| Number of people in the HH (T4) | 2.63 | 1.19 | 2 | 1 | 8 | 4,909 |
| Number of people in the HH (T5) | 2.60 | 1.18 | 2 | 1 | 8 | 4,679 |
| Number of people in the HH (T6) | 2.40 | 1.10 | 2 | 1 | 8 | 3,305 |
| Living in East Germany (T1) | 0.20 | 0.40 | 0 | 0 | 1 | 8,209 |
| Living in East Germany (T2) | 0.20 | 0.40 | 0 | 0 | 1 | 8,209 |
| Living in East Germany (T3) | 0.20 | 0.40 | 0 | 0 | 1 | 8,209 |
| Living in East Germany (T4) | 0.20 | 0.40 | 0 | 0 | 1 | 8,209 |
| Living in East Germany (T5) | 0.20 | 0.40 | 0 | 0 | 1 | 8,209 |
| Living in East Germany (T6) | 0.20 | 0.40 | 0 | 0 | 1 | 8,209 |
| Life satisfaction (T1) | 7.54 | 1.53 | 8 | 0 | 10 | 8,072 |
| Life satisfaction (T2) | 7.58 | 1.51 | 8 | 0 | 10 | 6,486 |
| Life satisfaction (T3) | 7.65 | 1.38 | 8 | 0 | 10 | 5,564 |
| Life satisfaction (T4) | 7.67 | 1.34 | 8 | 0 | 10 | 4,912 |
| Life satisfaction (T5) | 7.42 | 1.41 | 8 | 0 | 10 | 4,680 |
| Life satisfaction (T6) | 7.57 | 1.39 | 8 | 0 | 10 | 3,306 |
| Smoking (T1) | 0.30 | 0.46 | 0 | 0 | 1 | 6,081 |
| Smoking (T2) | 0.28 | 0.45 | 0 | 0 | 1 | 6,484 |
| Smoking (T3) | 0.25 | 0.43 | 0 | 0 | 1 | 5,563 |
| Smoking (T4) | 0.23 | 0.42 | 0 | 0 | 1 | 4,910 |
| Smoking (T5) | 0.22 | 0.41 | 0 | 0 | 1 | 4,679 |
| Smoking (T6) | 0.20 | 0.40 | 0 | 0 | 1 | 3,305 |
| Frequency alcohol consumption (T1) | 3.22 | 1.37 | 3 | 1 | 6 | 5,562 |
| Frequency alcohol consumption (T2) | 3.22 | 1.37 | 3 | 1 | 6 | 5,562 |
| Frequency alcohol consumption (T3) | 3.22 | 1.37 | 3 | 1 | 6 | 5,562 |
| Frequency alcohol consumption (T4) | 3.29 | 1.40 | 3 | 1 | 6 | 4,909 |
| Frequency alcohol consumption (T5) | 3.30 | 1.42 | 3 | 1 | 6 | 4,680 |
| Frequency alcohol consumption (T6) | 3.25 | 1.40 | 3 | 1 | 6 | 3,304 |
| Frequency doing sports (T1) | 3.02 | 1.18 | 3 | 1 | 5 | 5,562 |
| Frequency doing sports (T2) | 3.02 | 1.18 | 3 | 1 | 5 | 5,562 |
| Frequency doing sports (T3) | 3.02 | 1.18 | 3 | 1 | 5 | 5,562 |
| Frequency doing sports (T4) | 3.06 | 1.18 | 3 | 1 | 5 | 4,909 |
| Frequency doing sports (T5) | 3.12 | 1.20 | 3 | 1 | 5 | 4,679 |
| Frequency doing sports (T6) | 3.08 | 1.22 | 3 | 1 | 5 | 3,304 |

Source: NEPS SC6, observed (non-imputed) data of the analytic sample (N=8,209). Statistics are based on all non-missing values at each measurement point; N obs. gives the number of individuals with valid information. Frequency of alcohol consumption and sports participation at T1 and T2 were taken from T3 (see Methods) and are therefore identical to T3. Compare Table A1 for the corresponding statistics based on the imputed data. Observed mean BMI by sex, with 95% confidence intervals, is shown in Figure A1.

Figure A1: Mean BMI over time, by sex, observed cases only (no imputation)

Source: NEPS SC6, observed (non-imputed) data of the analytic sample; N varies by measurement point (see Table A7). 95% confidence intervals included. Graph is purely descriptive as no controls are included. Compare Figure 1 in the main text (imputed data).

Figure A2: BMI categories (WHO definition) for each survey point, by sex, observed cases only (no imputation)

Source: NEPS SC6, observed (non-imputed) data of the analytic sample; N varies by measurement point (see Table A7). Graph is purely descriptive as no controls are included. Compare Figure 2 in the main text (imputed data).

Table A8: FE regressions with measurement-point dummies instead of linear time

|  | M17 | M18 |
| --- | --- | --- |
|  | BMI (continuous) | Being overweight/obese |
| Log. HH income° | 0.102^**^ | 0.009 |
|  | [0.041,0.162] | [-0.001,0.018] |
| Employed | -0.145^**^ | -0.007 |
|  | [-0.254,-0.036] | [-0.025,0.010] |
| Unemployment episode reported | 0.070 | 0.007 |
|  | [-0.049,0.189] | [-0.010,0.025] |
| ISEI° | -0.058 | -0.009 |
|  | [-0.153,0.037] | [-0.021,0.004] |
| Living together with partner | 0.276^**^ | 0.031^*^ |
|  | [0.082,0.469] | [0.005,0.056] |
| Living together with children | -0.026 | 0.001 |
|  | [-0.142,0.090] | [-0.017,0.019] |
| Number of people in the HH | -0.017 | -0.000 |
|  | [-0.074,0.041] | [-0.009,0.008] |
| Living in East Germany | -0.159 | -0.044 |
|  | [-0.495,0.177] | [-0.102,0.014] |
| Life satisfaction° | 0.033^*^ | 0.002 |
|  | [0.003,0.063] | [-0.003,0.007] |
| Smoking | -0.515^***^ | -0.047^***^ |
|  | [-0.638,-0.392] | [-0.065,-0.028] |
| Frequency alcohol consumption° | 0.091^**^ | 0.008 |
|  | [0.025,0.156] | [-0.001,0.017] |
| Frequency doing sports° | -0.133^***^ | -0.010^**^ |
|  | [-0.181,-0.085] | [-0.017,-0.004] |
| T2 (2014/15) | 0.515^***^ | 0.038^***^ |
|  | [0.407,0.623] | [0.020,0.057] |
| T3 (2017/18) | 0.761^***^ | 0.060^***^ |
|  | [0.606,0.917] | [0.038,0.082] |
| T4 (2019/20) | 0.805^***^ | 0.069^***^ |
|  | [0.618,0.993] | [0.043,0.094] |
| T5 (2020/21) | 0.900^***^ | 0.080^***^ |
|  | [0.716,1.084] | [0.054,0.106] |
| T6 (2023/24) | 0.938^***^ | 0.082^***^ |
|  | [0.638,1.238] | [0.048,0.115] |
| Female respondent # T2 (2014/15) | 0.042 | -0.009 |
|  | [-0.090,0.175] | [-0.031,0.013] |
| Female respondent # T3 (2017/18) | -0.001 | -0.006 |
|  | [-0.168,0.165] | [-0.030,0.019] |
| Female respondent # T4 (2019/20) | -0.021 | -0.015 |
|  | [-0.255,0.213] | [-0.047,0.017] |
| Female respondent # T5 (2020/21) | -0.006 | -0.011 |
|  | [-0.239,0.227] | [-0.041,0.020] |
| Female respondent # T6 (2023/24) | 0.512^*^ | 0.034 |
|  | [0.045,0.979] | [-0.014,0.083] |
| Age at baseline (centred at 44) # T2 (2014/15) | -0.008 | -0.000 |
|  | [-0.016,0.000] | [-0.002,0.001] |
| Age at baseline (centred at 44) # T3 (2017/18) | -0.016^***^ | -0.001 |
|  | [-0.026,-0.007] | [-0.003,0.000] |
| Age at baseline (centred at 44) # T4 (2019/20) | -0.027^***^ | -0.002^*^ |
|  | [-0.037,-0.017] | [-0.004,-0.000] |
| Age at baseline (centred at 44) # T5 (2020/21) | -0.032^***^ | -0.003^**^ |
|  | [-0.043,-0.022] | [-0.004,-0.001] |
| Age at baseline (centred at 44) # T6 (2023/24) | -0.048^***^ | -0.004^***^ |
|  | [-0.061,-0.034] | [-0.006,-0.002] |
| Migration background # T2 (2014/15) | -0.095 | 0.002 |
|  | [-0.243,0.053] | [-0.022,0.026] |
| Migration background # T3 (2017/18) | -0.007 | 0.001 |
|  | [-0.175,0.162] | [-0.028,0.029] |
| Migration background # T4 (2019/20) | -0.020 | 0.002 |
|  | [-0.194,0.155] | [-0.027,0.032] |
| Migration background # T5 (2020/21) | -0.060 | -0.011 |
|  | [-0.252,0.131] | [-0.040,0.019] |
| Migration background # T6 (2023/24) | 0.046 | 0.003 |
|  | [-0.204,0.295] | [-0.031,0.036] |
| HEE (Abitur) # T2 (2014/15) | -0.128 | 0.010 |
|  | [-0.286,0.030] | [-0.016,0.036] |
| HEE (Abitur) # T3 (2017/18) | -0.061 | 0.013 |
|  | [-0.246,0.124] | [-0.015,0.042] |
| HEE (Abitur) # T4 (2019/20) | -0.053 | 0.006 |
|  | [-0.244,0.137] | [-0.024,0.037] |
| HEE (Abitur) # T5 (2020/21) | -0.122 | 0.002 |
|  | [-0.321,0.077] | [-0.029,0.034] |
| HEE (Abitur) # T6 (2023/24) | -0.095 | 0.005 |
|  | [-0.338,0.148] | [-0.029,0.039] |
| Tertiary # T2 (2014/15) | -0.059 | 0.007 |
|  | [-0.203,0.086] | [-0.018,0.032] |
| Tertiary # T3 (2017/18) | -0.125 | 0.006 |
|  | [-0.283,0.034] | [-0.021,0.032] |
| Tertiary # T4 (2019/20) | -0.210^*^ | -0.006 |
|  | [-0.376,-0.044] | [-0.034,0.021] |
| Tertiary # T5 (2020/21) | -0.248^**^ | -0.013 |
|  | [-0.426,-0.070] | [-0.042,0.015] |
| Tertiary # T6 (2023/24) | -0.238^*^ | 0.002 |
|  | [-0.445,-0.032] | [-0.030,0.034] |
| Social origin° # T2 (2014/15) | 0.021 | 0.006 |
|  | [-0.041,0.084] | [-0.005,0.017] |
| Social origin° # T3 (2017/18) | 0.013 | 0.000 |
|  | [-0.057,0.084] | [-0.013,0.013] |
| Social origin° # T4 (2019/20) | -0.011 | 0.001 |
|  | [-0.084,0.062] | [-0.012,0.013] |
| Social origin° # T5 (2020/21) | -0.001 | 0.001 |
|  | [-0.078,0.077] | [-0.012,0.014] |
| Social origin° # T6 (2023/24) | -0.023 | 0.002 |
|  | [-0.117,0.072] | [-0.012,0.016] |
| B5: Extraversion° # T2 (2014/15) | 0.018 | -0.000 |
|  | [-0.043,0.079] | [-0.011,0.011] |
| B5: Extraversion° # T3 (2017/18) | -0.004 | -0.000 |
|  | [-0.082,0.073] | [-0.012,0.012] |
| B5: Extraversion° # T4 (2019/20) | -0.000 | 0.000 |
|  | [-0.078,0.077] | [-0.011,0.012] |
| B5: Extraversion° # T5 (2020/21) | 0.014 | 0.003 |
|  | [-0.068,0.096] | [-0.010,0.015] |
| B5: Extraversion° # T6 (2023/24) | 0.075 | 0.011 |
|  | [-0.028,0.179] | [-0.003,0.026] |
| B5: Agreeableness° # T2 (2014/15) | -0.006 | 0.007 |
|  | [-0.061,0.050] | [-0.002,0.017] |
| B5: Agreeableness° # T3 (2017/18) | 0.020 | 0.010 |
|  | [-0.047,0.087] | [-0.001,0.020] |
| B5: Agreeableness° # T4 (2019/20) | 0.026 | 0.009 |
|  | [-0.048,0.100] | [-0.003,0.021] |
| B5: Agreeableness° # T5 (2020/21) | 0.031 | 0.012 |
|  | [-0.047,0.110] | [-0.000,0.024] |
| B5: Agreeableness° # T6 (2023/24) | 0.011 | 0.010 |
|  | [-0.091,0.112] | [-0.004,0.024] |
| B5: Conscientiousness° # T2 (2014/15) | -0.086^**^ | -0.002 |
|  | [-0.142,-0.030] | [-0.012,0.008] |
| B5: Conscientiousness° # T3 (2017/18) | -0.079^*^ | -0.003 |
|  | [-0.148,-0.010] | [-0.014,0.008] |
| B5: Conscientiousness° # T4 (2019/20) | -0.094^*^ | -0.006 |
|  | [-0.169,-0.019] | [-0.018,0.006] |
| B5: Conscientiousness° # T5 (2020/21) | -0.091^*^ | -0.002 |
|  | [-0.168,-0.014] | [-0.016,0.011] |
| B5: Conscientiousness° # T6 (2023/24) | -0.083 | 0.004 |
|  | [-0.175,0.010] | [-0.011,0.018] |
| B5: Neuroticism° # T2 (2014/15) | -0.013 | -0.002 |
|  | [-0.073,0.048] | [-0.013,0.009] |
| B5: Neuroticism° # T3 (2017/18) | -0.007 | -0.001 |
|  | [-0.083,0.069] | [-0.013,0.011] |
| B5: Neuroticism° # T4 (2019/20) | -0.013 | 0.002 |
|  | [-0.092,0.066] | [-0.012,0.015] |
| B5: Neuroticism° # T5 (2020/21) | -0.030 | -0.002 |
|  | [-0.110,0.051] | [-0.014,0.011] |
| B5: Neuroticism° # T6 (2023/24) | 0.019 | 0.005 |
|  | [-0.077,0.116] | [-0.009,0.020] |
| B5: Openness° # T2 (2014/15) | -0.039 | -0.002 |
|  | [-0.099,0.021] | [-0.011,0.008] |
| B5: Openness° # T3 (2017/18) | 0.010 | 0.000 |
|  | [-0.055,0.076] | [-0.010,0.011] |
| B5: Openness° # T4 (2019/20) | 0.039 | 0.004 |
|  | [-0.030,0.109] | [-0.008,0.016] |
| B5: Openness° # T5 (2020/21) | 0.034 | 0.000 |
|  | [-0.041,0.109] | [-0.011,0.012] |
| B5: Openness° # T6 (2023/24) | -0.012 | -0.003 |
|  | [-0.103,0.078] | [-0.016,0.010] |
| Self-worth° # T2 (2014/15) | 0.069 | 0.004 |
|  | [-0.003,0.140] | [-0.007,0.015] |
| Self-worth° # T3 (2017/18) | 0.012 | 0.004 |
|  | [-0.081,0.105] | [-0.010,0.019] |
| Self-worth° # T4 (2019/20) | 0.035 | 0.007 |
|  | [-0.051,0.121] | [-0.007,0.020] |
| Self-worth° # T5 (2020/21) | -0.031 | 0.003 |
|  | [-0.122,0.061] | [-0.011,0.017] |
| Self-worth° # T6 (2023/24) | 0.037 | 0.008 |
|  | [-0.075,0.148] | [-0.008,0.024] |
| Risk taking° # T2 (2014/15) | -0.040 | 0.004 |
|  | [-0.103,0.023] | [-0.007,0.015] |
| Risk taking° # T3 (2017/18) | 0.010 | 0.009 |
|  | [-0.064,0.084] | [-0.003,0.022] |
| Risk taking° # T4 (2019/20) | -0.011 | 0.005 |
|  | [-0.090,0.067] | [-0.008,0.019] |
| Risk taking° # T5 (2020/21) | -0.036 | 0.006 |
|  | [-0.124,0.053] | [-0.008,0.020] |
| Risk taking° # T6 (2023/24) | 0.003 | 0.008 |
|  | [-0.093,0.099] | [-0.007,0.023] |
| Mental health score° # T2 (2014/15) | -0.032 | -0.004 |
|  | [-0.098,0.035] | [-0.015,0.007] |
| Mental health score° # T3 (2017/18) | -0.035 | -0.009 |
|  | [-0.113,0.043] | [-0.022,0.005] |
| Mental health score° # T4 (2019/20) | -0.112^**^ | -0.013^*^ |
|  | [-0.195,-0.030] | [-0.026,-0.001] |
| Mental health score° # T5 (2020/21) | -0.104^*^ | -0.015^*^ |
|  | [-0.189,-0.018] | [-0.028,-0.001] |
| Mental health score° # T6 (2023/24) | -0.139^*^ | -0.019^**^ |
|  | [-0.245,-0.034] | [-0.033,-0.005] |
| Physical health score° # T2 (2014/15) | -0.050 | 0.004 |
|  | [-0.113,0.013] | [-0.007,0.014] |
| Physical health score° # T3 (2017/18) | -0.086^*^ | 0.008 |
|  | [-0.164,-0.009] | [-0.003,0.019] |
| Physical health score° # T4 (2019/20) | -0.126^**^ | 0.004 |
|  | [-0.206,-0.046] | [-0.007,0.016] |
| Physical health score° # T5 (2020/21) | -0.108^*^ | 0.001 |
|  | [-0.196,-0.019] | [-0.011,0.013] |
| Physical health score° # T6 (2023/24) | -0.020 | 0.017^*^ |
|  | [-0.130,0.089] | [0.002,0.032] |
| Constant | 26.096^***^ | 0.534^***^ |
|  | [25.855,26.337] | [0.497,0.571] |
| Observations | 49254 | 49254 |

Source: NEPS SC6, imputed data (N=8,209; M=50). 95% confidence intervals included, based on robust standard errors. Time is modelled with dummy variables for measurement points T2 to T6 (reference: T1, 2011/12); all time-constant variables are interacted with these dummies. Age at baseline is centred at 44 years; the measurement-point coefficients refer to a 44-year-old man with low education, no migration background, and average values on all standardized variables. ° Variable is z-standardized. * p < 0.05, ** p < 0.01, *** p < 0.001
